# Perceptual thresholds for differences in CT noise texture

**DOI:** 10.1117/1.JMI.11.3.035501

**Published:** 2024-05-09

**Authors:** Luuk J. Oostveen, Kirsten Boedeker, Daniel Shin, Craig K. Abbey, Ioannis Sechopoulos

**Affiliations:** a Radboud University Medical Center, Nijmegen, The Netherlands; b Canon Medical Systems Corporation, Los Angeles, California, United States; c University of California, Santa Barbara, Santa Barbara, California, United States; d University of Twente, Enschede, The Netherlands; e Dutch Expert Centre for Screening, Nijmegen, The Netherlands

**Keywords:** noise texture, computed tomography, noise power spectrum, perception

## Abstract

**Purpose:**

The average ($f_{\mathrm{av}}$) or peak ($f_{\text{peak}}$) noise power spectrum (NPS) frequency is often used as a one-parameter descriptor of the CT noise texture. Our study develops a more complete two-parameter model of the CT NPS and investigates the sensitivity of human observers to changes in it.

**Approach:**

A model of CT NPS was created based on its $f_{\text{peak}}$ and a half-Gaussian fit (σ) to the downslope. Two-alternative forced-choice staircase studies were used to determine perceptual thresholds for noise texture, defined as parameter differences with a predetermined level of discrimination performance (80% correct). Five imaging scientist observers performed the forced-choice studies for eight directions in the $f_{\text{peak}}/\sigma$-space, for two reference NPSs (corresponding to body and lung kernels). The experiment was repeated with 32 radiologists, each evaluating a single direction in the $f_{\text{peak}}/\sigma$-space. NPS differences were quantified by the noise texture contrast ($C_{\text{texture}}$), the integral of the absolute NPS difference.

**Results:**

The two-parameter NPS model was found to be a good representation of various clinical CT reconstructions. Perception thresholds for $f_{\text{peak}}$ alone are 0.2  lp/cm for body and 0.4  lp/cm for lung NPSs. For σ, these values are 0.15 and 2  lp/cm, respectively. Thresholds change if the other parameter also changes. Different NPSs with the same $f_{\text{peak}}$ or $f_{\mathrm{av}}$ can be discriminated. Nonradiologist observers did not need more $C_{\text{texture}}$ than radiologists.

**Conclusions:**

$f_{\text{peak}}$ or $f_{\mathrm{av}}$ is insufficient to describe noise texture completely. The discrimination of noise texture changes depending on its frequency content. Radiologists do not discriminate noise texture changes better than nonradiologists.

## 1 Introduction

The visual appearance of medical images is influenced by both their noise magnitude and noise texture. In fact, it is known that both can affect the detectability of small and low-contrast lesions.^1^ Although multiple factors affect noise magnitude, the noise texture in CT imaging is mainly influenced by the reconstruction method and reconstruction kernel used. In addition, with certain iterative CT reconstruction methods, the reconstructed images can appear to radiologists as nonnatural, i.e., plasticky or blotchy.^2^^–^^5^ This appearance is usually associated with a shift of the noise power spectrum (NPS) toward the lower frequencies compared with that of images obtained with filtered-back projection (FBP).^6^ This shift downward in the noise frequencies is mainly due to these algorithms achieving a reduction in the image noise by increasing the spatial correlation across voxels, especially in low-dose conditions. As expected, this increase in correlation can lead to a lowering of the spatial resolution of the image.^7^^,^^8^

Depending on the contrast, newly developed, deep-learning-based reconstruction (DLR) algorithms seem to be able to decouple this usual relationship between spatial resolution and noise texture from each other to a larger extent than that existing in current iterative reconstruction methods.^9^ This may allow for new opportunities to manipulate noise texture during reconstruction, improving the detectability of low-contrast lesions.

Therefore, with the increasing use of iterative reconstruction algorithms in CT and especially with the advent of deep-learning-based postprocessing, it is of interest to better understand the phenomenon of noise texture changes during reconstruction and postprocessing in CT. With this knowledge, it could be feasible to tune some of these algorithms to optimize the resulting image noise texture while maintaining the spatial resolution. This can be achieved by studying only the shape of the NPS, independently of its magnitude. However, to make these insights clinically relevant, it is necessary to first determine what changes in noise texture are actually perceptible by a human observer. Given the complexity of the human visual system, it is not immediately clear how sensitive humans are to noise texture differences. Therefore, it is of interest to characterize the minimum changes in the NPS shape that are needed for a human observer to detect a change in the image texture.

To be able to systematically study noise texture changes, a simple and continuous parametric model that describes the NPS change, and therefore noise texture, is needed. It is common to summarize the information of CT NPSs with one parameter, the frequency at which the NPS peaks ($f_{\text{peak}}$), or alternatively, the average NPS frequency ($f_{\mathrm{av}}$). However, it is clear that one parameter can provide only limited information on the frequency distribution of the noise texture. In other words, multiple different NPSs, all resulting in different noise textures, could have the same $f_{\text{peak}}$ and/or $f_{\mathrm{av}}$. To overcome this, a more complete parametric representation of the CT NPS shape is needed.

Therefore, the purpose of this study is to introduce and validate a more complete parametric model of the NPS in CT and use that model to determine the detectability of changes in noise texture for human observers.

## 2 Materials and Methods

To investigate the perceptual thresholds for noise texture changes, we created and evaluated a simple and continuous two-parameter model that describes the shape of the NPS of CT images. This model was then used in forced-choice psychophysical experiments using adaptive staircase methods to estimate the observer thresholds as a function of changes in the two parameters. To understand if these perceptual thresholds might be different for radiologists compared with nonradiologists, a limited version of the study was repeated with radiologists. Finally, to determine if the threshold changes varied based on differences in the reference NPS, these experiments were performed using two different reference noise textures, one for body and the other for lung reconstruction kernels.

### 2.1 Modeling the Noise Texture

In CT, an NPS usually has a ramp dominating the lower frequencies and an apodization part dominating the higher frequencies.^10^ Previously, to model the full NPS, a six-parameter model of NPS was suggested:^11^

$$\mathrm{NPS}(f)=a\cdot f^{b}\cdot e^{-\frac{|f^{c}-\alpha {|}^{d}}{2\beta^{2}}}.$$ (1)

In this model, the parameter a controls the magnitude of the noise, and the other parameters primarily determine the shape of the NPS. However, having six parameters that can change alone or together results in a large number of possible changes and is impractical for use in an observer study. Therefore, we propose the simplification of the model to a three-parameter one. By evaluating the resulting NPS fits from one manufacturer, the values of b, c, and d were empirically determined and fixed to 1 for b and c and 2 for d:

$$\mathrm{NPS}(f)=a\cdot f\cdot e^{-\frac{(f-\alpha {)}^{2}}{2\beta^{2}}}.$$ (2)

The applicability of this model for clinically available reconstruction kernels and reconstruction methods in CT for various vendors is tested.

If an NPS is described using Eq. (2), the peak frequency of this NPS is derived analytically as follows:

$$f_{\text{peak}}=\frac{\alpha +\sqrt{\alpha^{2}+4\beta^{2}}}{2}.$$ (3)

**Fig. 1:**
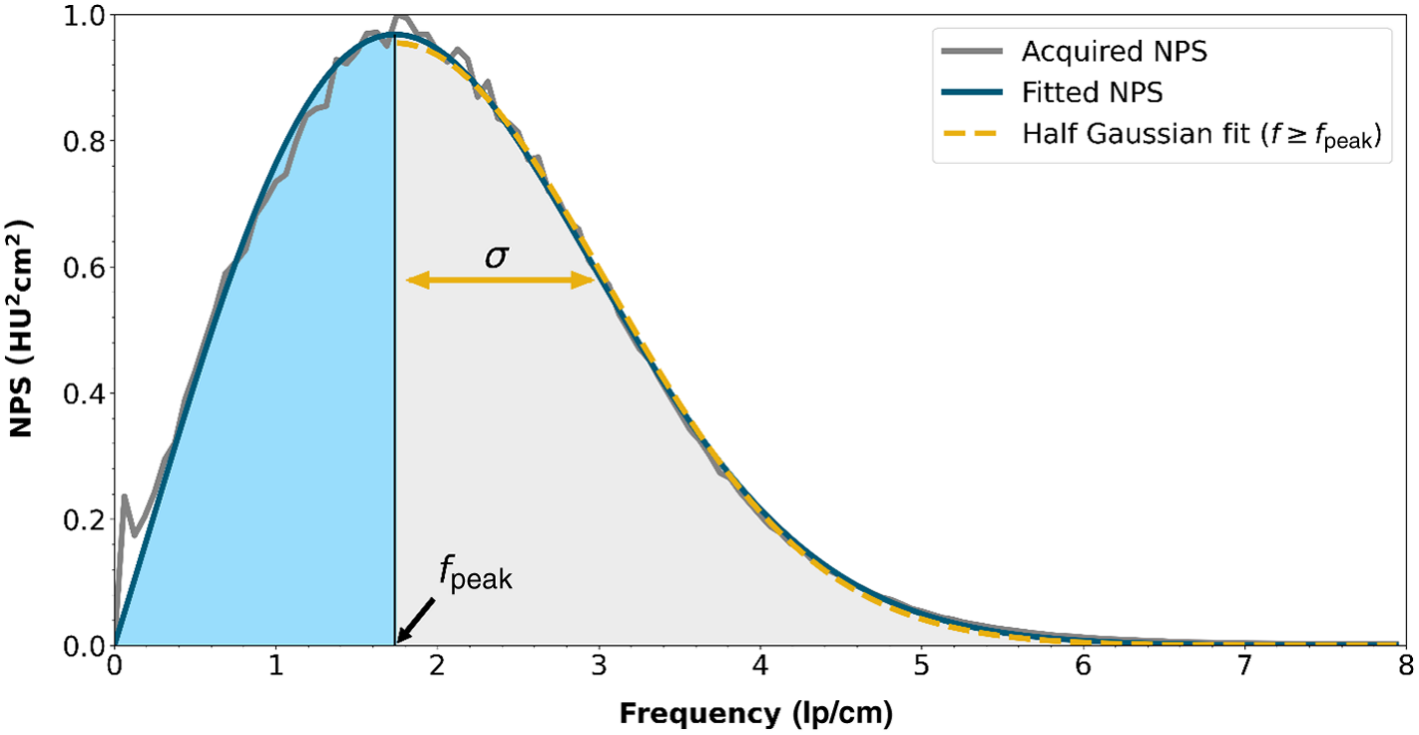
Example of NPS parameterization. The original acquired NPS (in gray) is fit using the three-parameter model [Eq. (2), in dark blue]. The peak frequency ($f_{\text{peak}}$) is used to describe the first section of the NPS (in light blue). A half-Gaussian is fit through the section beyond $f_{\text{peak}}$ [Eq. (4), dashed yellow]. The σ of that Gaussian describes the apodization part of the NPS (light gray).

To characterize the NPS independently of the fitting model used, we propose two parameters: one parameter that describes the upslope and one that describes the downslope. For the description of the upslope, we used $f_{\text{peak}}$ because NPSs are supposed to monotonically increase to $f_{\text{peak}}$ and this parameter is already often used to describe the NPS. For the downslope, we used the standard deviation (σ) of a half-Gaussian that is fitted through the downslope of the NPS, i.e., for all frequencies equal to or higher than $f_{\text{peak}}$, resulting in

$$g(f)=a^{\prime }\cdot e^{-\frac{{\left(f-f_{\text{peak}}\right)}^{2}}{2\sigma^{2}}}\;f\geq f_{\text{peak}},$$ (4)

where $a^{\prime }$ determines the magnitude of the Gaussian and σ is its width. So σ was used as a single parameter to describe the NPS downslope. Because we are modeling only the shape of the NPS and not its overall magnitude, all modeled NPSs were set to unit area under the curve. An example NPS and its resulting parameterizations are shown in Fig. 1.

Information on the testing on the applicability of this model is given in Appendix A.

### 2.2 Generation of Patches with Various Noise Textures

Given a specific $f_{\mathrm{peak}}$ and σ, a continuous distribution of NPSs can be generated using Eq. (1) or Eq. (2). For a detailed description of the procedure used, see Appendix A. From the NPS resulting from these equations, a two-dimensional NPS (${\mathrm{NPS}}_{2D}$) was created assuming that the ${\mathrm{NPS}}_{2D}$ is radially symmetric. To be able to generate a specific noise texture, the generated ${\mathrm{NPS}}_{2D}$ is applied to white noise as follows:

$$N(\mu ,\sigma )=F^{-1}\{\sqrt{{\mathrm{NPS}}_{2D}}\cdot F\{n(\mu =0,\sigma =1)\}\},$$ (5)

where N is the resulting colored-noise image, F is the FFT operator, and n is a realization of white Gaussian noise with a mean value of μ and a standard deviation of σ. For the observer study, noise patches of 256×256  pixels were created.

### 2.3 Noise Texture Contrast

If two noise textures and their corresponding NPSs are considered, the noise texture contrast ($C_{\text{texture}}$) is calculated based on the contrast that an ideal observer is able to see. For the derivation of the ideal observer, see Appendix B. Effectively, the ideal observer looks at the absolute differences between the two NPSs. Therefore, the noise texture contrast is calculated from the ${\mathrm{NPS}}_{2D}$ as

$$C_{\text{texture}}=\sum |{\mathrm{NPS}}_{2D,1}(f,\omega )-{\mathrm{NPS}}_{2D,2}(f,\omega )|.$$ (6)

### 2.4 Observer Study

**Fig. 2:**
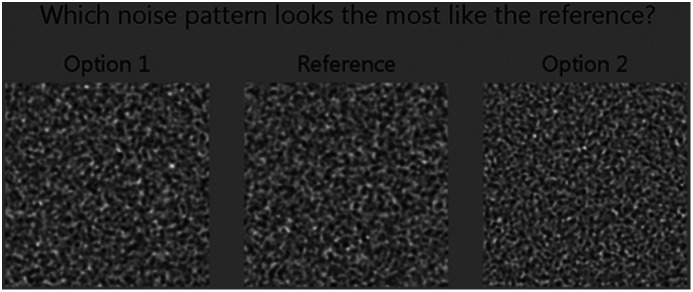
Screenshot of a trial shown to the observer. The task of the observer was to select the alternative noise patch (option 1 or option 2) that has the noise texture most comparable to the one of the reference noise patch.

To investigate the detectability of differences between two colored noise textures, a two alternative forced choice observer study was performed. For each realization of the experiment, three noise patches, created in real time, were shown to the observer. One was labeled the “reference” noise patch, one patch had another noise realization with the same NPS as the reference patch, and the third noise patch originated from an NPS with a different $f_{\text{peak}}$ and/or a different σ. All noise patches were shown with a window level equal to the mean gray level and a window width of 10 (i.e., 10 times the SD). The task for the observer was to identify the patch that had the same noise texture as the reference noise patch (Fig. 2). After the observer made a choice, the correct patch was highlighted for one second and then the next trial was shown.

To determine the parameter values to use for the noise patches of the next trial, a staircase method was applied with a step size of 15% of the current value. The difference in the parameter values between the different NPSs was decreased after three correct responses and increased after one incorrect response.^12^ The trials were stopped after 12 reversals, and every series was executed 6 times. Per repetition, the geometrical mean over the trials from the last eight reversals was determined, which is an estimate of the 80% correct point on the psychometric curve.^13^ The average value of this 80% correct point from the last five repetitions was used as the detectability threshold.

**Fig. 3:**
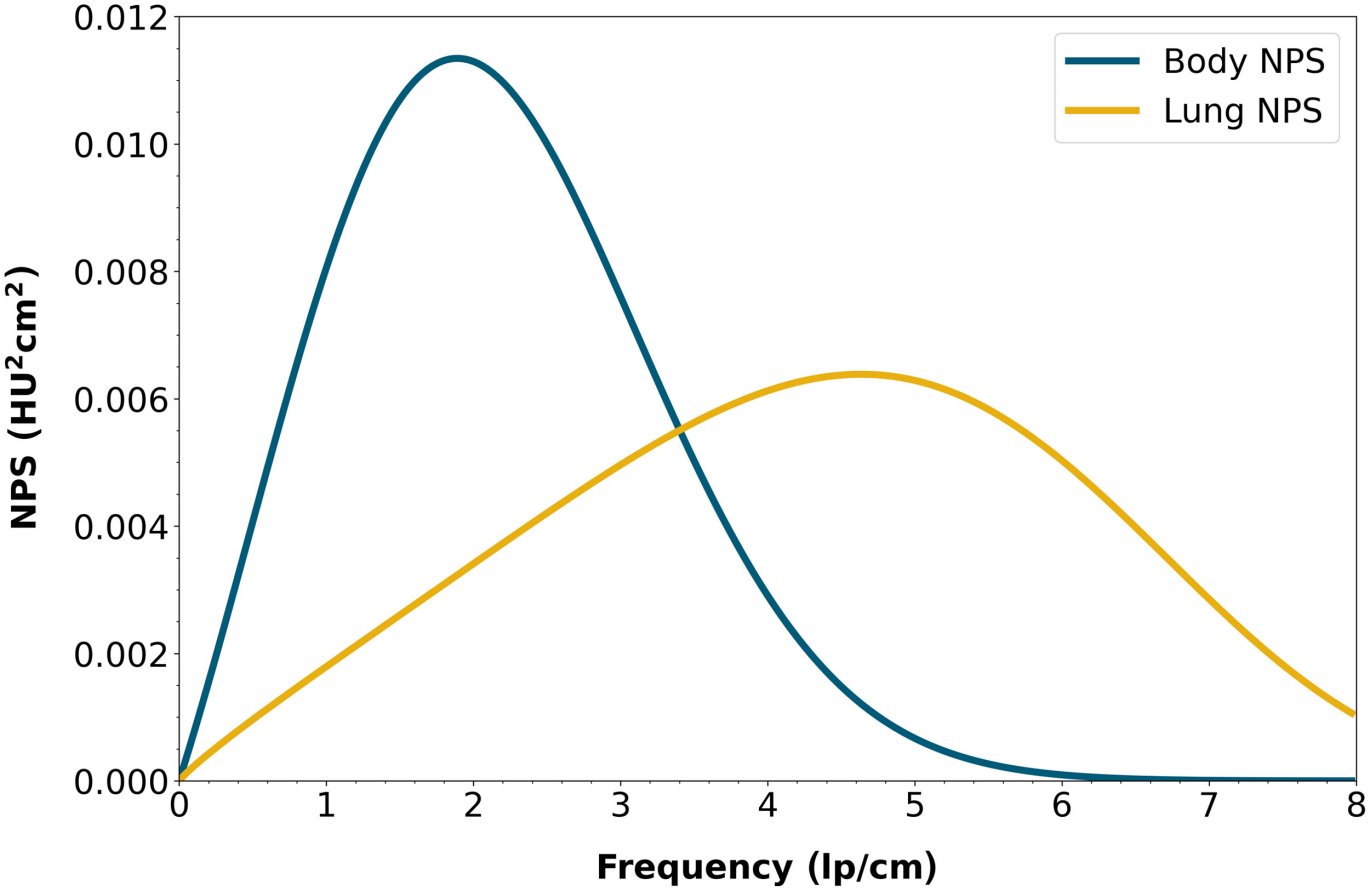
Reference noise power spectra (NPSs). The body NPS was obtained using an HIR (Hybrid-IR) method with body settings, and for the lung NPS, a Hybrid-IR with lung settings was used. The $f_{\text{peak}}$ and σ values are 1.89 and 1.28  lp/cm for the body kernel and 4.64 and 1.83  lp/cm for the lung kernel.

For this study, two reference NPSs were chosen, one from a body kernel and one resulting from a lung kernel, determined from images of a 320 mm water phantom on a clinical wide-area CT system (Aquilion One PRISM edition, Canon Medical Systems Corporation, Otawara, Japan) using the dose determined by the automatic tube current modulation and a hybrid iterative reconstruction (HIR) method (AIDR 3D, Canon Medical Systems Corporation). The reference NPSs are shown in Fig. 3. The $f_{\text{peak}}$ and σ values are 1.89 and 1.28  lp/cm for the body kernel and 4.64 and 1.83  lp/cm for the lung kernel.

**Fig. 4:**
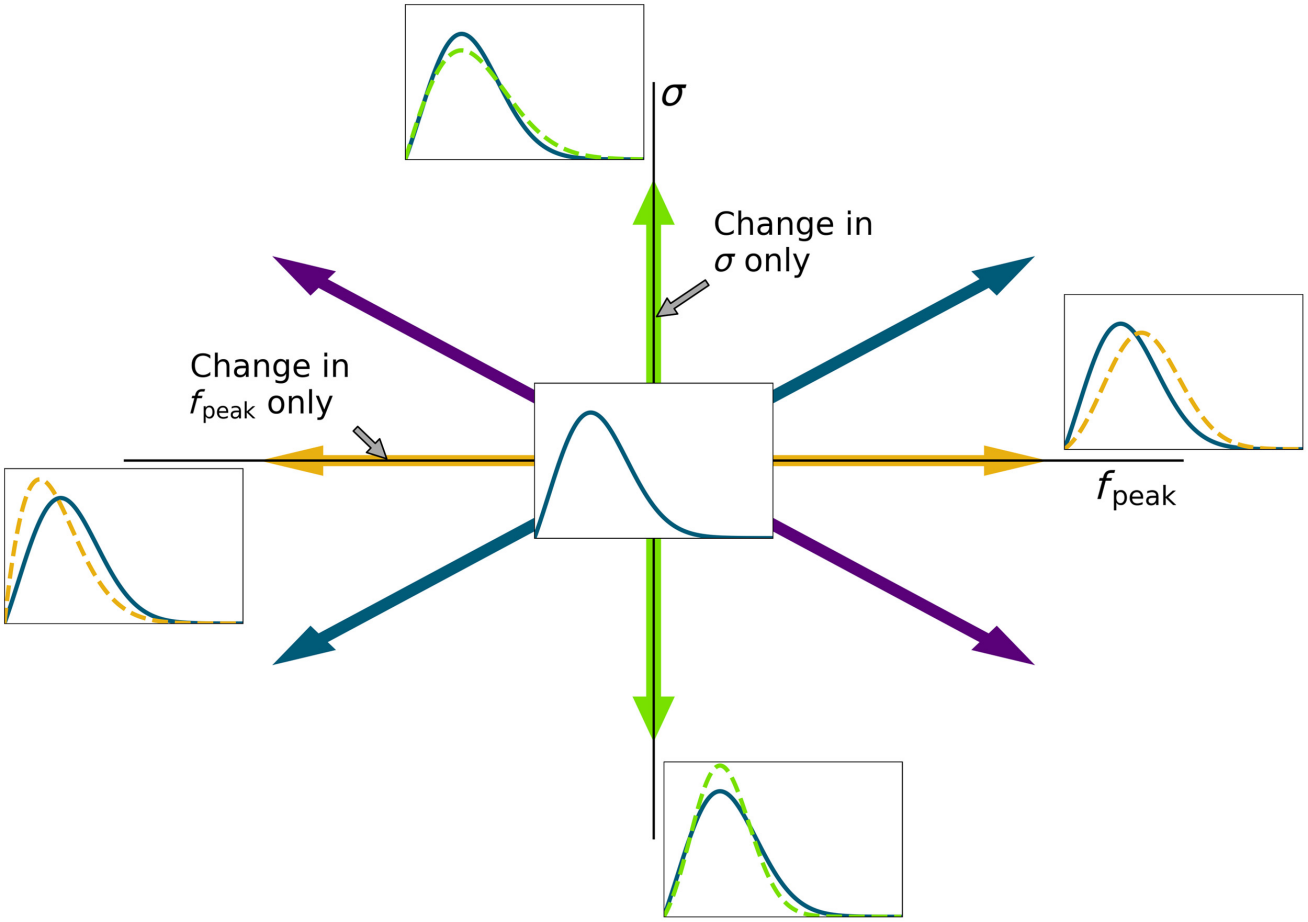
All eight directions of changes in $f_{\text{peak}}$ and/or σ that were investigated. Compared to the reference NPS, four directions change $f_{\text{peak}}$ or σ only, and four directions involve a change in both $f_{\text{peak}}$ and σ.

During the observer study, the two reference NPSs were approached from eight directions, involving a change in $f_{\text{peak}}$ only, a change in σ only, and simultaneous changes in both (all from higher and lower values) (see Fig. 4). The starting test values were determined by what was a clearly visible difference in noise texture for one of the investigators. Initially, five nonradiologist observers (PhD students in imaging science and medical physics trainees) completed studies to evaluate all 8 directions for both reference NPSs in multiple sessions. A maximum of 2 directions was performed in one session to prevent fatigue. All observers were able to complete each two-direction session within 1 h.

To investigate if radiologists are able to detect more subtle differences in noise texture, the experiment was repeated with 27 radiologists at the Medical Imaging Perception Lab at the European Congress of Radiology (ECR) 2023 and afterward with five radiologists from the Radboud University Medical Center. Due to the limitation in available time per radiologist, each radiologist only performed one of the reference NPS and direction combinations. Each series was performed only five times, of which the last four were used for the calculation of the geometrical mean. This led to results from two radiologists for each direction.

All experiments were performed in dimmed lighting conditions, comparable to diagnostic reading room conditions. The noise patches were shown on a DICOM GSDF calibrated diagnostic monitor (for the nonradiologist: Barco MDMC-12133 and for the radiologists: Barco MDNC-3321, Barco, Kortrijk, Belgium).

### 2.5 Analysis of the Results

For each reference NPS, the threshold $f_{\text{peak}}$ and σ values per nonradiologist observer and the average threshold over all nonradiologist observers were calculated for each direction. A threshold detectability boundary ellipse was fitted through the eight average threshold values using a least squares method.

The threshold noise texture contrast for each threshold condition, as well as their 95% confidence interval, was calculated per observer. For the two radiologists, only the limiting noise texture contrast was calculated. The radiologists were assumed to perform the same as the other observers if their threshold noise texture contrast was within the 95% confidence interval of the nonradiologist observers.

## 3 Results

**Fig. 5:**
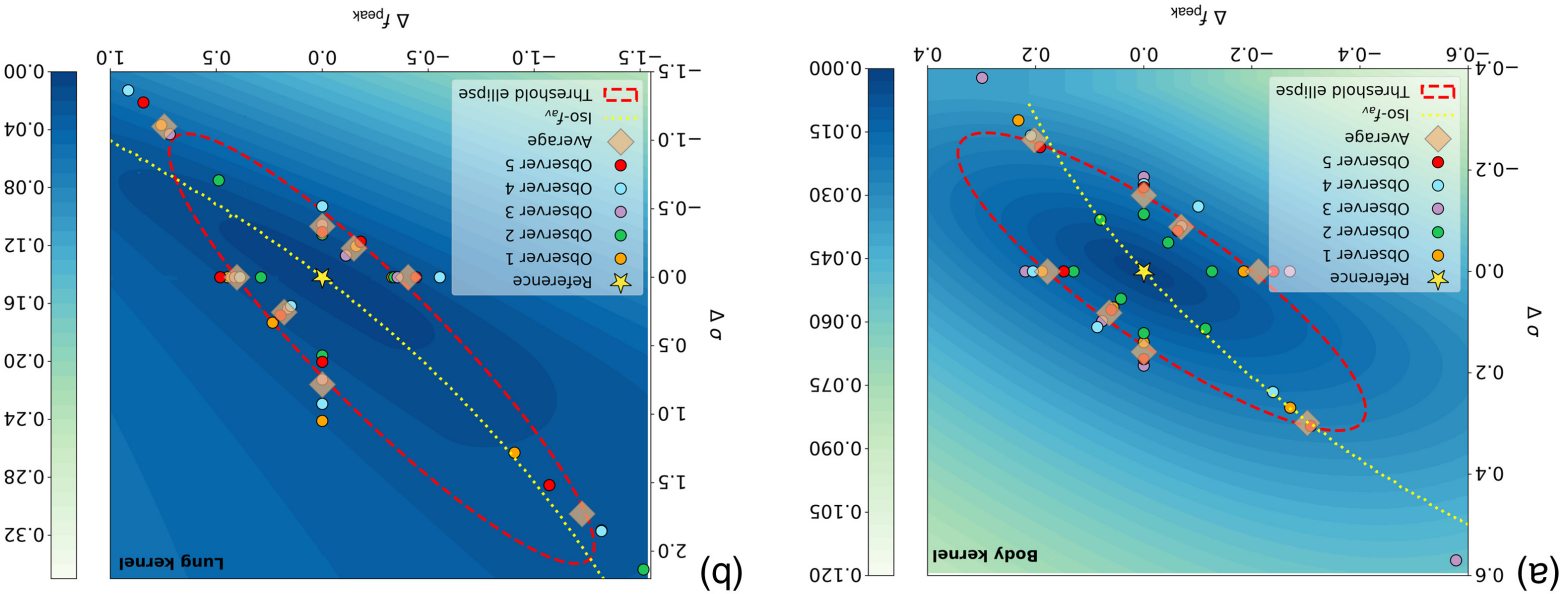
Results from the observer study. The 80% threshold limits for each observer, as well as the average values and the fitted detectability threshold ellipse, are shown. Results from the (a) body reference NPS and (b) lung reference NPS. The color of the background indicates the noise texture contrast.

In Fig. 5, the $f_{\text{peak}}$ and σ limiting values for each nonradiologist observer and the overall average are shown for both reference NPSs. Also the detectability threshold ellipse is shown. For the body NPS ($f_{\text{peak}}$: 1.89  lp/cm and σ: 1.28  lp/cm), the ellipse has the center close to the reference value, with $f_{\text{peak}}=1.86\;\mathrm{lp}/\mathrm{cm}$ and σ=1.30  lp/cm. The major radius of the ellipse makes an angle of 143 deg with the $f_{\text{peak}}$ axis. Based on this elliptical fit, the detectability threshold $f_{\text{peak}}$ is 0.2  lp/cm. Of course, this value changes if σ is changed simultaneously. For the lung NPS ($f_{\text{peak}}$: 4.64  lp/cm and σ: 1.83  lp/cm), the ellipse center is at $f_{\text{peak}}=4.30\;\mathrm{lp}/\mathrm{cm}$ and σ=2.46  lp/cm, and the major radius makes an angle of 120 deg. The corresponding threshold $f_{\text{peak}}$ is 0.4  lp/cm. Therefore, the detection threshold for a change in $f_{\text{peak}}$ is higher when using the lung NPS as the reference compared with the body NPS as reference.

The background of the two graphs in Fig. 5 shows the noise texture contrast ($C_{\text{texture}}$) compared to the reference NPS. The lighter the color is, the higher the contrast is. For a changing $f_{\text{peak}}$, less $C_{\text{texture}}$ is needed to be perceptible to a human observer compared with changing the downslope. To make a change in texture perceptible, the most $C_{\text{texture}}$ is needed in the direction of lowering $f_{\text{peak}}$ combined with increasing σ, or vice versa. The iso-$f_{\mathrm{av}}$ line through the reference NPS shows that the average frequency is a better estimator for the visibility of noise texture changes than $f_{\text{peak}}$ because the iso-$f_{\mathrm{av}}$ line is more parallel to the major axis of the threshold ellipse, whereas the iso-$f_{\text{peak}}$ line runs more closely to the minor axis of the ellipse. However, NPSs with the same average frequency can still be distinguishable from each other.

**Fig. 6:**
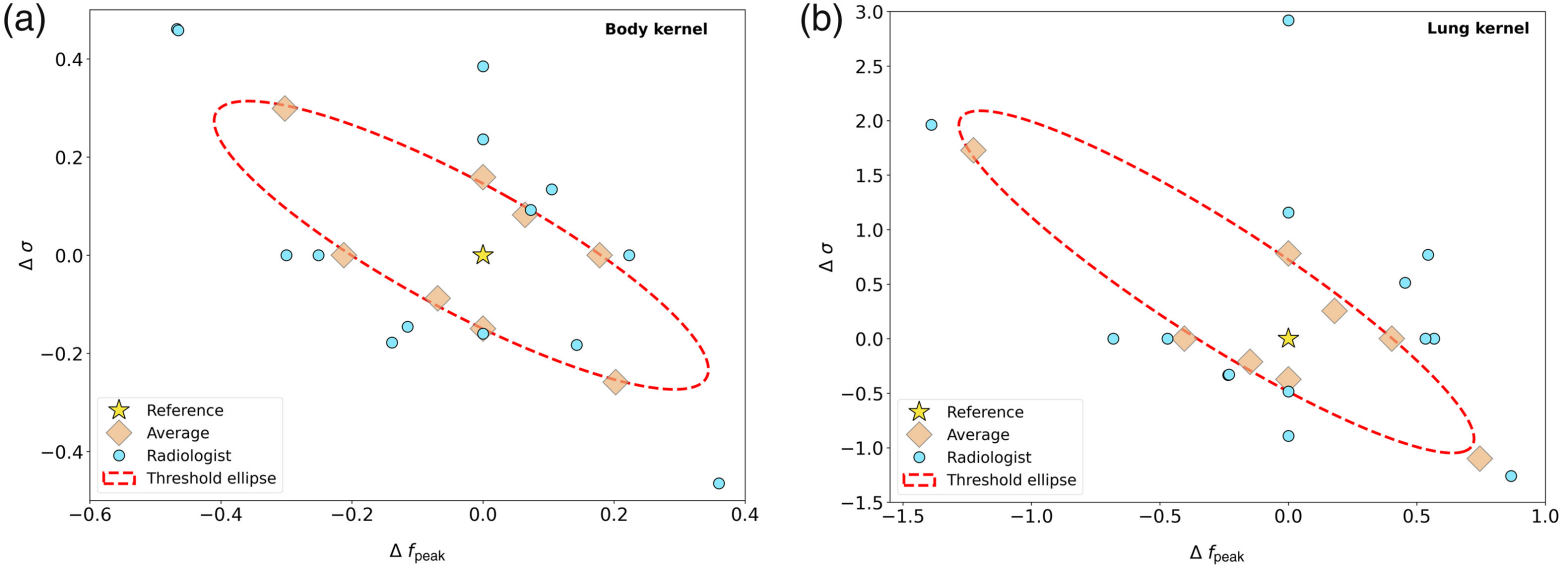
(a), (b) Results from the radiologists shown in conjunction with the average of the nonradiologist observers. In 17 of the 32 radiologist experiments, the results are within the 95% confidence intervals of the nonradiologist results.

The noise texture contrast thresholds determined with radiologists show that radiologists have a noise texture contrast threshold within the 95% confidence interval of the nonradiologist observer results in 17 of the 32 experiments. In 12 cases, the radiologists had a noise texture contrast threshold above the 95% confidence interval of that from the nonradiologist observers. A detectability threshold could not be determined for two experiments because the observers would have needed a larger difference to be able to detect the correct noise texture than would be possible (σ would become negative). Table 1 and Fig. 6 show the individual radiologist results and the average results of the nonradiologist observers.

**Table 1:** 80% detection thresholds for both body and lung NPS as a reference for the nonradiologist observers. Also the noise texture contrast ($C_{\text{texture}}$) for the first and second radiologists is shown. If the $C_{\text{texture}}$ of the radiologist is in bold, this value is outside the 95% interval of the nonradiologist observers.

Direction	Body					Lung				
	$\Delta f_{\text{peak}}$ (lp/cm)	Δσ (lp/cm)	$C_{\text{texture}}$ (${\mathrm{HU}}^{2}$)	First radiologist $C_{\text{texture}}$ (${\mathrm{HU}}^{2}$)	Second radiologist $C_{\text{texture}}$ (${\mathrm{HU}}^{2}$)	$\Delta f_{\text{peak}}$ (lp/cm)	Δσ (lp/cm)	$C_{\text{texture}}$ (${\mathrm{HU}}^{2}$)	First radiologist $C_{\text{texture}}$ (${\mathrm{HU}}^{2}$)	Second radiologist $C_{\text{texture}}$ (${\mathrm{HU}}^{2}$)
1	0.18 ± 0.07	n.a.	0.014 ± 0.006	**0.034**	0.018	0.40 ± 0.14	n.a.	0.019 ± 0.006	**0.025**	0.024
2	−0.21 ± 0.11	n.a.	0.017 ± 0.009	0.023	0.020	−0.40 ± 0.19	n.a.	0.019 ± 0.009	**0.033**	0.022
3	n.a.	0.16 ± 0.05	0.020 ± 0.006	0.022	^a^	n.a.	0.78 ± 0.40	0.025 ± 0.008	0.031	**0.045**
4	n.a.	-0.15 ± 0.07	0.020 ± 0.010	**0.045**	0.029	n.a.	-0.37 ± 0.17	0.023 ± 0.013	**0.072**	0.032
5	0.06 ± 0.03	0.08 ± 0.04	0.015 ± 0.008	**0.024**	0.017	0.18 ± 0.07	0.26 ± 0.10	0.017 ± 0.005	**0.032**	**0.038**
6	−0.07 ± 0.04	−0.09 ± 0.05	0.017 ± 0.010	**0.035**	0.028	−0.15 ± 0.07	−0.21 ± 0.10	0.019 ± 0.010	**0.033**	0.032
7	0.20 ± 0.16	−0.26 ± 0.20	0.026 ± 0.020	0.041	0.041	0.75 ± 0.32	−1.10 ± 0.50	0.047 ± 0.031	0.056	0.056
8	−0.30 ± 0.33	0.30 ± 0.33	0.027 ± 0.031	0.036	0.018	−1.23 ± 0.47	1.73 ± 0.65	0.026 ± 0.007	0.028	^a^

n.a.: not applicable (in this direction, this parameter does not change compared to the reference).

a No value available for the second radiologist for directions 3 (abdomen) and 8 (lung) because, for these observers, the difference had to be larger than possible for σ (σ would become negative).

## 4 Discussion

Because noise texture influences detectability of lesions and new deep learning-based CT methods can more easily modify noise texture, it is of interest to study the effect of noise texture changes on the detectability of lesions. In this research, we focused on the detectability of noise texture changes itself, hypothesizing that if an observer cannot detect differences in noise texture, then lesion detectability across these noise textures would be unaffected. We found the thresholds for detectability with varying $f_{\text{peak}}$ and σ for two commonly used reconstruction kernels and showed that radiologists do not perform better than nonradiologist observers in detecting these differences. This may suggest that the sensitivity to changes in NPS is related to the human visual system. However, depending on the direction of the change, the intraobserver variability of this detectability threshold can be large. This is especially true in the direction of the major axis of the ellipse.

The change in $f_{\text{peak}}$ and σ needed for the human observer to detect the difference varies for both conditions and for the directions within each condition. However, the average noise-texture contrast needed is roughly equivalent, except for the lung reference NPS in the direction of a higher $f_{\text{peak}}$ and lower σ. In this case, the noise texture differences are concentrated at high spatial frequencies. This result may reflect limitations in human observers at high spatial frequencies. This would be consistent with models of texture discrimination that posit octave bandwidth filters that are broad band at higher spatial frequencies and therefore mix noise effects across a larger band of spatial frequencies.^14^^–^^16^ However, we should note that these models have been developed for images that look very different from CT noise textures. Therefore, further validation is needed to determine if generalization to CT noise is applicable.

A slightly higher detectability threshold was found for radiologists compared with the nonradiologist observers. This might be caused by the fact that the evaluation with radiologists was performed in only one change direction, so they were less used to the task compared with the nonradiologists, who did all directions. In addition, radiologists only performed five repetitions, and the nonradiologist observers performed six. However, we did not find that the last repetition of the nonradiologist observers was better than the first five.

This research is a first step in the investigation of the effect of noise texture from nonlinear reconstruction methods on the perception of lesions in clinical CT images. Further research is needed to include the visibility of lesions with different noise textures and, eventually, with the inclusion of anatomical background. The latter is needed not only due to its interference with the detectability of lesions but also because the noise texture from nonlinear reconstructions is probably different from that in homogeneous backgrounds, potentially also breaking the assumption that the noise is radially symmetrical. However, in this first initial study, we aimed to determine what differences in noise texture, as characterized by differences in NPS, are actually detectable by the human visual system, so the follow-up studies could be performed with meaningful noise texture differences.

Our study has several limitations. First, the number of observers was limited. Future research might involve increasing the number of observers to better estimate the average thresholds as well as their variability for the various directions. Also evaluating only eight directions of change is quite limited considering that six parameters are needed to describe an ellipse. Hence, evaluating more directions could provide a better estimation of the limiting ellipse. In addition, we used only two reference NPSs. Although these NPSs are used often for lung and body exams, acquisitions for bone and brain result in NPSs having different $f_{\text{peak}}$ and σ. Also other reconstruction techniques, such as model-based iterative reconstructions (MBIR) or DLR, as well as reconstructions from other vendors, will result in different NPS shapes. Finally, the underlying noise distribution used was Gaussian, although in recent studies, we are seeing that nonnormal CT noise distributions can be discriminable from NPS-matched normal noise distributions.^17^ To further study this effect, similar studies as this one are needed; however, these should not change the NPS but change the underlying noise distribution. Next, just as a follow-up for this study, the effect on lesion detection should be studied.

## 5 Conclusions

Human observers showed different sensitivity to changes in CT noise textures based on peak frequency ($f_{\text{peak}}$), and the downslope of the NPS (σ) alone and in combination. Radiologists did not detect these textural changes any better than nonradiologist observers. Describing NPS using only the $f_{\text{peak}}$ or the $f_{\mathrm{av}}$ alone was insufficient to describe perceived differences in CT noise texture. The presented model using $f_{\text{peak}}$ and σ can serve as a starting point to better describe noise texture and to further study the impact of CT noise texture on human task performance.

## 6 Appendix A: Verification of the NPS Model in CT

To obtain a wide representation of CT NPS curves, water phantom images were acquired using CTs from four vendors and different reconstruction techniques. These NPSs were modeled and parameterized, and the goodness-of-fit for the models and appropriateness of the parameters was determined.

### 6.1 NPS Acquisition

NPS data were acquired on four CT systems from four different vendors (Canon Medical Systems, GE HealthCare, Philips healthcare, Siemens Healthineers). A 320-mm diameter water phantom was imaged using the settings used clinically for the abdomen protocol at the corresponding site and with a lower dose setting. The acquisitions were reconstructed using the clinically used kernel for abdomen, lung, brain, and bone, for the following reconstruction methods (if available): FBP, HIR, MBIR, and DLR. The slice thicknesses used were 0.5 mm for Canon, 0.625 mm for GE, and 1.0 mm for Philips and Siemens. For all but the FBP reconstruction, three strength settings were used, leading to a maximum of 80 reconstructions per CT system, if all reconstruction methods were available [2 dose levels, 4 kernels, 10 reconstruction methods (1 FBP + 3 strengths × 3 methods)]. Each ${\mathrm{NPS}}_{1d}$ was calculated in a central ROI of 128×128  pixels using the method described by Boedeker et al.^18^ From each acquisition, a stack of at least 100 slices was used for the NPS calculation. For each slice, the NPS was calculated and averaged over all slices. This average ${\mathrm{NPS}}_{1d}$ was normalized to have a unit area under the curve.

### 6.2 NPS Data Analysis

The acquired ${\mathrm{NPS}}_{1d}$ was fitted using the six-parameter model [Eq. (1)] and the three-parameter model [Eqs. (2) and (3)] by a least-squares method. To determine the $f_{\text{peak}}$, the ${\mathrm{NPS}}_{1d}$ was first filtered using a low-pass filter at 4% of the full bandwidth. This prevented small local peaks from affecting the determination of $f_{\text{peak}}$. Finally, the three-parameter ${\mathrm{NPS}}_{1d}$ was described with a two-parameter model [with Eq. (4)], and the $f_{\mathrm{av}}$ was calculated.

To generate the two-parameter parameterized NPS, a procedure in Python was written using the curve_fit and minimize_scalar functions from the scipy.optimize package. The three parameterized NPSs were compared to the original ${\mathrm{NPS}}_{1d}$ using the relative sum of absolute differences (RSAD):

$$\mathrm{RSAD}=\underset{f}{\sum }\frac{\left|{\mathrm{NPS}}_{\mathrm{param}}(f)-{\mathrm{NPS}}_{1d}(f)\right|}{{\mathrm{NPS}}_{1d}(f)}\times 100\%.$$ (7)

### 6.3 Results

All acquired NPS shapes are shown in Sec. 6.4. Of the 194 acquired NPSs, 152 (78%) NPSs have a shape that has a ramp dominating the low frequencies and an apodization part that dominates the higher frequencies. 14 (7%) NPSs only have a ramp (no downslope), and 28 (14%) NPSs have a different shape altogether (e.g., multiple peaks).

**Fig. 7:**
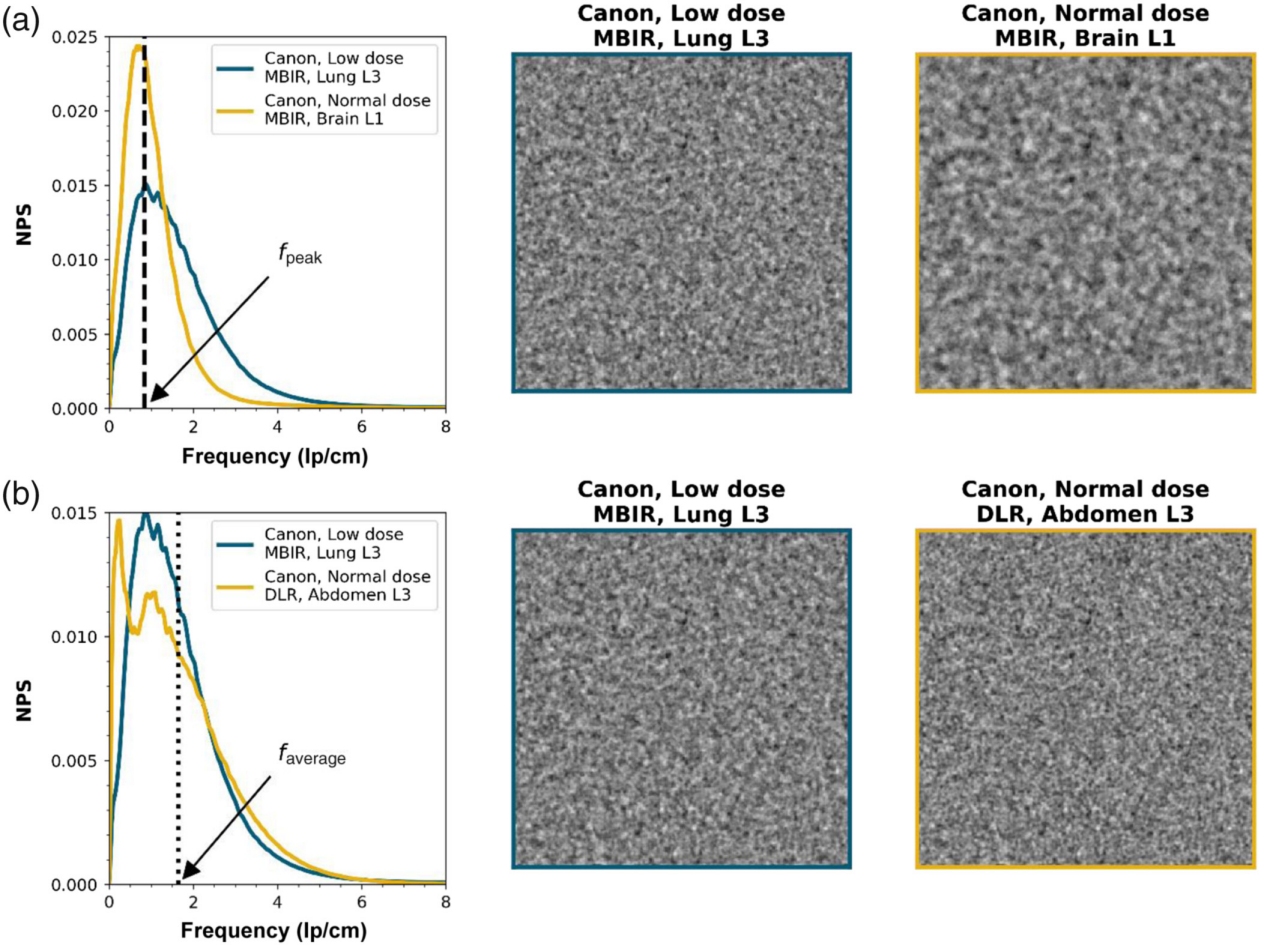
NPSs with clearly different noise textures but with the same (a) peak frequency (1.26  lp/cm) and (b) average peak frequency (1.64  lp/cm). To avoid possible differences in appearance due to differences in higher order statistics in the original reconstructions, the noise textures were generated by applying the NPS to a realization of white noise.

In Tables 2–5, all values of $f_{\text{peak}}$, σ, $f_{\mathrm{av}}$, and the RSADs between the parameterized NPSs and the acquired NPSs are given. For several NPSs, there is no σ value because the NPS has no downslope within the Nyquist frequency. Two NPSs with the same $f_{\text{peak}}$ or the same $f_{\mathrm{av}}$ are shown in Fig. 7 together with their corresponding noise textures. As can be seen, the noise textures are clearly discernible, whereas $f_{\text{peak}}$ or $f_{\mathrm{av}}$ is the same. The combination of $f_{\text{peak}}$ and σ do differ for these situations.

**Table 2:** Parameters and appropriateness of fit of the various NPSs acquired on a Canon Aquilion One PRISM Edition.

	Canon	$f_{\text{peak}}$ (lp/cm)	σ (lp/cm)	$f_{\mathrm{av}}$ (lp/cm)	Normal dose					$f_{\text{peak}}$ (lp/cm)	σ (lp/cm)	$f_{\mathrm{av}}$ (lp/cm)	Low dose				
					Six-param. model		Three-param. model		Modeled NPS				Six-param. model		Three-param. model		Modeled NPS
					$R^{2}$	Diff. (%)	$R^{2}$	RSAD (%)	Diff. (%)				$R^{2}$	RSAD (%)	$R^{2}$	RSAD (%)	RSAD (%)
FBP	Abdomen	2.63	1.52	2.89	1.00	1.23	1.00	2.57	3.55	2.66	1.51	2.92	1.00	2.37	1.00	2.74	3.65
	Lung	7.59	—	5.69	1.00	1.20	1.00	1.38	33.79	7.59	-	5.71	1.00	1.58	1.00	1.23	34.57
	Brain	2.38	2.32	3.24	1.00	1.04	1.00	2.24	3.09	2.47	2.29	3.25	1.00	1.07	1.00	2.03	1.97
	Bone	6.09	3.89	5.37	1.00	1.97	1.00	1.73	19.96	6.81	2.45	5.39	1.00	1.82	1.00	1.56	14.02
HIR	Abdomen-L1	2.03	1.34	2.32	1.00	1.44	1.00	2.73	4.92	1.56	1.20	1.91	1.00	1.73	1.00	3.36	3.26
	Abdomen-L2	1.91	1.30	2.21	1.00	1.63	1.00	2.86	5.05	1.47	1.16	1.82	1.00	1.88	1.00	3.30	3.32
	Abdomen-L3	1.66	1.20	1.97	1.00	1.93	1.00	3.09	5.33	1.31	1.00	1.59	1.00	2.17	1.00	3.75	5.58
	Lung-L1	4.19	1.75	3.96	1.00	1.00	0.98	6.33	16.92	2.59	1.67	2.87	1.00	1.14	1.00	3.17	7.26
	Lung-L2	3.53	2.05	3.72	1.00	1.21	0.98	5.21	6.45	2.03	1.81	2.65	1.00	1.33	1.00	2.13	2.22
	Lung-L3	2.75	2.36	3.43	1.00	1.68	0.99	3.64	4.94	1.75	1.72	2.41	1.00	1.67	1.00	2.40	3.28
	Brain-L1	1.91	1.49	2.35	1.00	1.27	1.00	2.37	3.67	1.47	1.26	1.92	1.00	1.61	1.00	2.81	2.83
	Brain-L2	1.84	1.44	2.28	1.00	1.30	1.00	2.75	4.40	1.44	1.22	1.87	1.00	1.72	1.00	3.10	3.07
	Brain-L3	1.59	1.32	2.06	1.00	1.73	1.00	3.34	3.49	1.31	1.08	1.68	1.00	2.11	1.00	3.68	4.07
	Bone-L1	4.88	1.50	4.52	1.00	2.28	0.99	7.05	18.30	3.56	1.48	3.46	1.00	1.43	0.99	5.92	14.70
	Bone-L2	4.56	1.66	4.33	1.00	1.84	0.98	7.51	13.50	3.03	1.72	3.26	1.00	1.23	0.99	5.79	6.00
	Bone-L3	4.38	1.73	4.09	1.00	1.13	0.97	7.85	18.10	2.75	1.77	3.02	1.00	1.21	0.99	5.05	7.43
MBIR	Abdomen-L1	1.78	1.41	2.29	1.00	4.14	1.00	5.60	10.36	1.34	1.25	1.91	1.00	3.85	1.00	6.55	6.75
	Abdomen-L2	1.47	1.30	2.04	1.00	4.67	1.00	6.52	8.60	1.13	1.12	1.70	1.00	3.84	1.00	7.76	7.47
	Abdomen-L3	1.13	1.18	1.75	1.00	4.91	1.00	7.58	7.31	0.91	0.96	1.45	1.00	3.99	1.00	9.12	8.67
	Lung-L1	2.38	1.80	2.91	1.00	3.96	0.99	5.21	6.78	2.09	1.40	2.40	1.00	5.57	0.99	5.83	13.45
	Lung-L2	1.84	1.47	2.31	1.00	4.96	0.99	5.43	11.48	1.50	1.36	2.03	1.00	4.00	1.00	4.57	8.22
	Lung-L3	1.31	1.33	1.90	1.00	3.45	1.00	4.26	8.59	0.84	1.25	1.64	1.00	3.65	1.00	6.73	7.91
	Brain-L1	0.84	0.83	1.27	1.00	4.20	1.00	8.08	7.16	0.69	0.65	1.06	1.00	5.29	1.00	10.39	9.74
	Brain-L2	0.69	0.65	1.06	1.00	4.83	0.99	10.53	10.43	0.56	0.51	0.89	1.00	7.55	0.99	12.65	16.44
	Brain-L3	0.50	0.49	0.84	1.00	9.39	0.99	12.57	14.96	0.44	0.38	0.70	1.00	11.79	0.99	15.08	27.49
	Bone-L1	6.09	2.92	5.05	1.00	0.85	1.00	1.96	6.07	6.06	3.25	5.06	1.00	1.43	1.00	1.98	1.97
	Bone-L2	4.56	2.84	4.47	1.00	1.68	1.00	2.03	2.94	3.63	2.11	3.77	0.99	3.93	0.99	4.79	10.25
	Bone-L3	2.38	1.91	3.01	1.00	3.90	0.99	4.84	5.22	2.13	1.55	2.60	1.00	4.98	1.00	5.41	9.31
DLR	Abdomen-L1	1.91	1.72	2.37	1.00	2.32	0.98	6.40	14.80	1.25	1.56	1.91	1.00	3.49	0.96	9.97	9.03
	Abdomen-L2	1.22	1.85	2.14	0.99	3.55	0.96	9.19	9.36	1.19	1.41	1.73	1.00	3.69	0.95	12.79	11.80
	Abdomen-L3	0.34	1.85	1.64	0.98	7.41	0.89	16.81	87.07	0.34	1.52	1.35	0.97	9.85	0.87	22.21	70.89
	Lung-L1	4.03	1.79	3.83	0.99	3.91	0.94	8.97	16.52	2.81	1.62	2.97	1.00	2.23	0.97	8.35	10.75
	Lung-L2	3.56	2.02	3.55	0.91	8.60	0.78	13.73	16.41	2.06	1.91	2.60	0.99	3.27	0.93	12.60	11.17
	Lung-L3	0.31	4.48	3.12	0.89	9.68	0.61	16.71	144.53	1.25	1.61	2.01	1.00	2.02	0.99	5.42	5.15
	Brain-L1	1.72	1.21	2.02	1.00	2.11	1.00	3.44	6.00	1.38	1.10	1.73	1.00	2.21	1.00	3.62	3.64
	Brain-L2	1.66	1.16	1.95	1.00	2.28	1.00	3.85	5.78	1.38	1.09	1.72	1.00	2.30	1.00	3.93	4.04
	Brain-L3	1.66	1.12	1.91	1.00	2.36	1.00	4.11	6.94	1.38	1.08	1.72	1.00	2.38	1.00	4.15	4.32
	Bone-L1	4.13	1.74	3.75	1.00	1.26	0.93	10.29	25.23	3.28	1.67	3.16	1.00	1.66	0.96	10.38	21.86
	Bone-L2	3.50	1.97	3.33	1.00	1.49	0.86	14.68	25.56	1.16	2.54	2.55	0.99	3.73	0.95	12.77	26.81
	Bone-L3	0.59	3.33	2.73	0.97	6.81	0.83	19.50	91.19	1.13	1.94	2.13	0.99	5.47	0.99	7.02	7.39

RSAD: Relative sum of absolute differences between the acquired and the modeled NPS. Modeled NPS: NPS determined based on the two-parameter model, using $f_{\text{peak}}$ and σ from the acquired NPS.

**Table 3:** Parameters of the various NPSs acquired on a GE Discovery CT750 HD.

	GE	$f_{\text{peak}}$ (lp/cm)	σ (lp/cm)	$f_{\mathrm{av}}$ (lp/cm)	Normal dose					$f_{\text{peak}}$ (lp/cm)	σ (lp/cm)	$f_{\mathrm{av}}$ (lp/cm)	Low dose				
					six-param. model		Three-param. model		Modeled NPS				six-param. model		Three-param. model		Modeled NPS
					$R^{2}$	RSAD (%)	$R^{2}$	RSAD (%)	RSAD (%)				$R^{2}$	RSAD (%)	$R^{2}$	RSAD (%)	RSAD (%)
FBP	Abdomen	2.59	1.69	2.78	0.87	7.77	0.80	9.76	11.10	2.59	1.70	2.78	0.87	6.94	0.81	9.70	11.25
	Lung	5.00	1.56	4.78	1.00	1.89	0.99	4.28	9.39	5.06	1.52	4.79	1.00	1.88	0.99	4.50	12.27
	Brain	3.25	1.78	3.24	0.89	5.90	0.84	8.46	15.18	3.28	1.77	3.24	0.90	6.05	0.84	8.44	16.35
	Bone	7.63	—	5.50	0.98	7.90	0.99	5.57	28.90	7.63	—	5.52	0.98	8.27	0.99	5.71	29.96
HIR	Abdomen-L1	2.53	1.61	2.59	0.81	10.47	0.72	10.72	18.13	1.91	1.96	2.59	0.82	11.00	0.73	10.81	15.41
	Abdomen-L2	1.81	1.62	2.21	0.72	15.55	0.58	14.04	14.75	1.81	1.62	2.21	0.72	15.55	0.58	14.14	14.95
	Abdomen-L3	1.78	1.29	1.90	0.66	20.30	0.48	17.30	23.98	1.78	1.28	1.90	0.66	20.48	0.48	17.63	24.42
	Lung-L1	4.81	1.64	4.65	1.00	2.02	0.99	4.10	7.41	4.91	1.58	4.65	1.00	1.98	0.99	4.33	11.68
	Lung-L2	4.31	1.82	4.35	0.99	1.94	0.99	3.19	3.97	4.38	1.79	4.36	0.99	1.94	0.99	3.31	3.97
	Lung-L3	3.94	1.86	4.06	0.99	2.92	0.99	2.71	2.88	3.91	1.88	4.05	0.99	2.97	0.99	2.71	3.29
	Brain-L1	2.63	2.01	3.03	0.85	7.76	0.78	8.60	8.77	2.59	2.04	3.03	0.85	7.67	0.78	8.59	8.82
	Brain-L2	1.88	2.03	2.62	0.77	10.88	0.67	10.71	11.79	1.88	2.03	2.61	0.77	11.06	0.67	10.80	11.49
	Brain-L3	1.81	1.65	2.27	0.73	14.08	0.60	13.37	14.60	1.84	1.63	2.27	0.74	14.45	0.60	13.54	15.29
	Bone-L1	7.63	-	5.33	0.97	8.30	0.98	7.22	22.25	7.63	—	5.35	0.99	3.74	0.98	7.48	23.29
	Bone-L2	7.66	-	4.94	0.93	7.24	0.91	10.27	11.59	7.63	—	4.96	0.93	7.53	0.91	10.64	12.19
	Bone-L3	7.66	-	4.55	0.78	7.22	0.74	10.99	21.45	7.66	—	4.57	0.78	7.36	0.74	11.40	18.40

RSAD: Relative sum of absolute differences between the acquired and the modeled NPS. Modeled NPS: NPS determined based on the two-parameter model, using $f_{\text{peak}}$ and σ from the acquired NPS.

**Table 4:** Parameters of the various NPSs acquired on a Philips iCT 256.

	Philips	$f_{\text{peak}}$ (lp/cm)	σ (lp/cm)	$f_{\mathrm{av}}$ (lp/cm)	Normal dose					$f_{\text{peak}}$ (lp/cm)	σ (lp/cm)	$f_{\mathrm{av}}$ (lp/cm)	Low dose				
					six-param. model		Three-param. model		Modeled NPS				six-param. model		Three-param. model		Modeled NPS
					$R^{2}$	RSAD (%)	$R^{2}$	RSAD (%)	RSAD (%)				$R^{2}$	RSAD (%)	$R^{2}$	RSAD (%)	RSAD (%)
FBP	Abdomen	2.56	2.01	3.06	1.00	2.19	0.99	4.30	4.48	2.56	2.04	3.05	0.99	3.11	0.98	5.50	5.54
	Lung	5.44	1.41	4.77	1.00	1.87	0.97	10.29	32.06	5.47	1.40	4.79	1.00	2.26	0.96	10.71	31.86
	Brain	2.34	1.62	2.66	1.00	1.91	0.99	4.25	5.63	2.41	1.60	2.69	1.00	1.86	0.99	5.01	6.17
	Bone	5.44	1.41	4.77	1.00	1.87	0.97	10.29	32.06	6.00	1.23	5.27	1.00	3.74	0.98	10.59	34.11
HIR	Abdomen-L1	1.97	2.19	2.86	1.00	2.37	0.99	4.20	7.24	1.91	2.13	2.75	1.00	2.70	0.99	4.74	5.45
	Abdomen-L2	1.97	2.13	2.77	1.00	2.75	0.99	4.29	4.09	1.81	2.08	2.66	1.00	3.11	0.99	3.63	3.50
	Abdomen-L3	1.88	2.05	2.63	1.00	3.35	0.99	4.11	6.44	1.78	1.95	2.47	0.99	4.35	0.97	6.21	10.43
	Lung-L1	5.28	1.53	4.63	1.00	1.10	0.95	10.78	30.02	5.34	1.50	4.54	1.00	1.17	0.94	10.84	37.35
	Lung-L2	5.41	1.44	4.59	1.00	1.24	0.93	12.48	38.66	5.41	1.46	4.49	1.00	1.43	0.92	12.21	43.14
	Lung-L3	5.38	1.46	4.59	1.00	2.81	0.93	12.86	37.20	5.34	1.51	4.48	0.99	3.45	0.90	13.08	39.77
	Brain-L1	1.94	1.70	2.48	1.00	2.21	0.99	4.11	4.21	1.91	1.62	2.39	1.00	2.75	0.99	5.10	5.15
	Brain-L2	1.84	1.69	2.39	1.00	2.58	0.99	4.23	4.15	1.81	1.60	2.29	1.00	3.12	0.99	5.36	6.62
	Brain-L3	1.75	1.61	2.25	1.00	3.15	0.99	4.64	7.24	1.69	1.53	2.15	0.99	3.80	0.99	5.70	9.93
	Bone-L1	6.00	1.22	5.21	1.00	2.61	0.98	10.11	38.41	5.97	1.27	5.15	1.00	2.75	0.97	10.17	37.99
	Bone-L2	6.00	1.22	5.19	1.00	3.49	0.97	11.29	38.63	6.03	1.22	5.13	1.00	3.57	0.96	11.69	43.36
	Bone-L3	6.00	1.21	5.21	1.00	3.42	0.97	11.92	38.24	6.03	1.21	5.15	1.00	2.21	0.96	12.13	41.97
MBIR	Abdomen-L1	0.53	2.82	2.46	0.96	9.81	0.72	43.05	77.03	0.53	1.40	1.87	0.93	11.95	0.86	46.14	49.45
	Abdomen-L2	0.50	1.15	1.73	0.96	10.94	0.90	44.49	45.56	0.53	0.76	1.34	0.91	25.22	0.88	37.97	40.49
	Abdomen-L3	0.47	0.71	1.00	0.97	12.60	0.96	21.25	23.22	0.34	0.73	0.85	0.95	13.42	0.91	27.32	28.04
	Lung-L1	6.03	1.28	4.70	0.93	9.61	0.83	17.46	56.95	5.84	1.50	4.22	−2.37	18.54	−0.03	22.85	63.32
	Lung-L2	5.91	1.38	4.29	−1.77	21.04	0.06	23.85	65.66	0.44	8.78	3.62	−0.55	20.04	−2.44	35.01	124.60
	Lung-L3	0.44	1.55	2.05	0.96	8.71	0.84	54.82	65.83	0.31	0.91	1.44	0.97	9.48	0.84	53.82	57.10
	Brain-L1	0.38	2.14	1.89	0.96	14.64	0.77	34.73	84.70	0.47	1.51	1.58	0.97	11.21	0.88	34.56	50.12
	Brain-L2	0.34	1.29	1.65	0.98	12.52	0.82	65.51	65.86	0.44	0.64	1.30	0.96	12.76	0.93	41.99	42.21
	Brain-L3	0.34	0.75	1.16	0.98	9.27	0.91	44.35	40.50	0.44	0.54	0.98	0.96	14.09	0.95	28.56	33.53

RSAD: Relative sum of absolute differences between the acquired and the modeled NPS. Modeled NPS: NPS determined based on the two-parameter model, using $f_{\text{peak}}$ and σ from the acquired NPS.

**Table 5:** Parameters of the various NPSs acquired on a Siemens Somatom Force.

	Siemens	$f_{\text{peak}}$ (lp/cm)	σ (lp/cm)	$f_{\mathrm{av}}$ (lp/cm)	Normal dose					$f_{\text{peak}}$ (lp/cm)	σ (lp/cm)	$f_{\mathrm{av}}$ (lp/cm)	Low dose				
					six-param. model		Three-param. model		Modeled NPS				six-param. model		Three-param. model		Modeled NPS
					$R^{2}$	RSAD (%)	$R^{2}$	RSAD (%)	RSAD (%)				$R^{2}$	RSAD (%)	$R^{2}$	RSAD (%)	RSAD (%)
FBP	Abdomen	2.37	1.63	2.73	1.00	1.15	1.00	1.61	3.71	2.02	1.60	2.52	1.00	0.89	1.00	1.14	2.79
	Lung	4.68	1.66	4.52	1.00	1.97	1.00	1.83	4.26	4.18	1.62	4.18	1.00	2.04	1.00	2.00	2.33
	Brain	1.90	1.61	2.47	1.00	1.24	1.00	2.35	2.51	1.78	1.47	2.29	1.00	1.06	1.00	1.96	2.01
	Bone	7.13	-	5.51	1.00	1.37	1.00	4.05	44.99	7.11	-	5.08	1.00	1.40	1.00	1.72	21.34
MBIR	Abdomen-L1	1.99	1.81	2.66	1.00	1.13	1.00	1.69	6.50	1.96	1.59	2.46	1.00	1.10	1.00	1.54	1.75
	Abdomen-L2	1.78	1.77	2.47	1.00	1.63	1.00	2.11	3.38	1.70	1.58	2.27	1.00	1.51	1.00	2.33	2.21
	Abdomen-L3	1.32	1.60	2.11	1.00	2.40	0.99	5.48	5.36	1.29	1.39	1.93	1.00	5.73	1.00	5.38	5.91
	Lung-L1	4.39	1.86	4.48	1.00	2.03	1.00	1.99	8.21	4.21	1.60	4.13	1.00	2.15	1.00	2.08	6.49
	Lung-L2	4.30	1.90	4.30	1.00	2.13	1.00	3.17	4.07	3.80	1.82	3.93	1.00	1.90	1.00	2.99	3.39
	Lung-L3	3.92	2.05	3.72	1.00	1.59	0.97	4.85	17.24	2.49	2.44	3.31	1.00	2.24	0.99	3.39	7.21
	Brain-L1	1.84	1.57	2.40	1.00	1.13	1.00	2.73	2.58	1.78	1.40	2.22	1.00	1.22	1.00	2.91	3.59
	Brain-L2	1.73	1.42	2.22	1.00	1.26	1.00	4.28	4.47	1.55	1.33	2.06	1.00	1.46	1.00	4.14	4.16
	Brain-L3	1.58	1.21	1.98	1.00	1.76	1.00	5.39	5.78	1.46	1.14	1.86	1.00	1.86	1.00	4.32	4.37
	Bone-L1	7.13	—	5.47	1.00	1.45	1.00	4.61	43.51	7.11	8.34	5.02	1.00	1.12	1.00	1.85	20.07
	Bone-L2	7.13	—	5.30	0.98	9.09	0.99	6.74	37.80	7.11	5.82	4.79	0.99	3.61	0.99	4.95	13.18
	Bone-L3	7.11	—	4.64	0.84	16.62	0.80	21.73	28.70	7.08	—	4.01	0.55	9.45	0.22	23.07	30.50

RSAD: relative sum of absolute differences between the acquired and the modeled NPS. Modeled NPS: NPS determined based on the two-parameter model, using $f_{\text{peak}}$, and σ from the acquired NPS.

The six-parameter NPS model can fit the acquired NPSs with a RSAD smaller than 10% for FBP, HIR, MBIR, and DLR in 100%, 92%, 71%, and 100% of the cases, respectively. For the three-parameter model, this drops to 88%, 69%, 45%, and 54%, respectively. For a more elaborate overview, see Table 6. The NPSs modeled by the parameters $f_{\text{peak}}$ and σ using the three-parameter model yielded a modeled NPS that, over all manufacturers, resulted within 20% RSAD for FBP, HIR, MBIR, and DLR in 69%, 78%, 54%, and 67% of all NPSs, respectively (Table 6).

**Table 6:** Percentages of NPS parameterizations with a relative sum of absolute differences with the acquired NPS below 10%, between 10% and 20%, and above 20%, per manufacturer and reconstruction type. The first number in each cell is the percentage for the six-parameter model and the second number is for the Three-parameter model. Some reconstruction types were not available for some manufacturers.

Reconstruction type	Deviation	Manufacturer				
		Canon	GE	Philips	Siemens	All
FBP	<10%	100/100	100/100	100/50.0	100/100	100/87.5
	>10%, <20%	0/0	0/0	0/50.0	0/0	0/12.5
	>20%	0/0	0/0	0/0	0/0	0/0
HIR	<10%	100/100	75.0/41.7	100/50.0	Not available	91.7/68.9
	>10%, <20%	0/0	16.7/58.3	0/50.0		5.2/28.9
	>20%	0/0	8.3/0	0/0		3.1/2.1
MBIR	<10%	100/79.2	Not available	33.3/0	91.7/91.7	71.4/45.2
	>10%, <20%	0/20.8		50.0/5.6	4.2/0	21.4/14.3
	>20%	0/0		16.7/94.4	4.2/8.3	7.1/40.5
DLR	<10%	100/54.2	Not available	Not available	Not available	100/54.2
	>10%, <20%	0/41.7				0/41.7
	>20%	0/4.2				0/4.2

### 6.4 NPS Shapes

NPS shapes for the images obtained for the various manufacturers, reconstruction techniques, and kernels. The acquired NPS and the fitted three-parameter model are shown below. 

**Figure:**
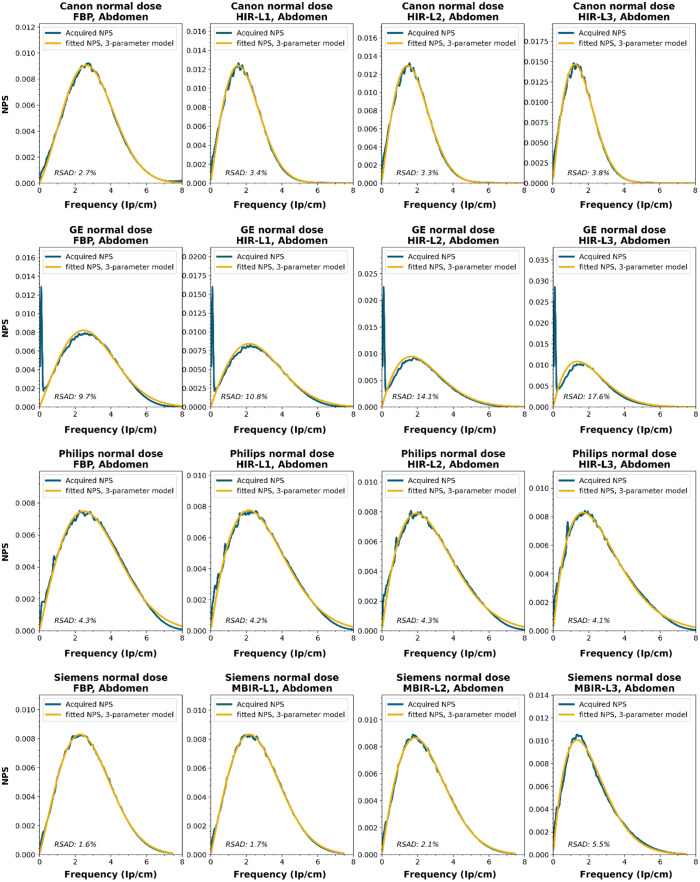


**Figure:**
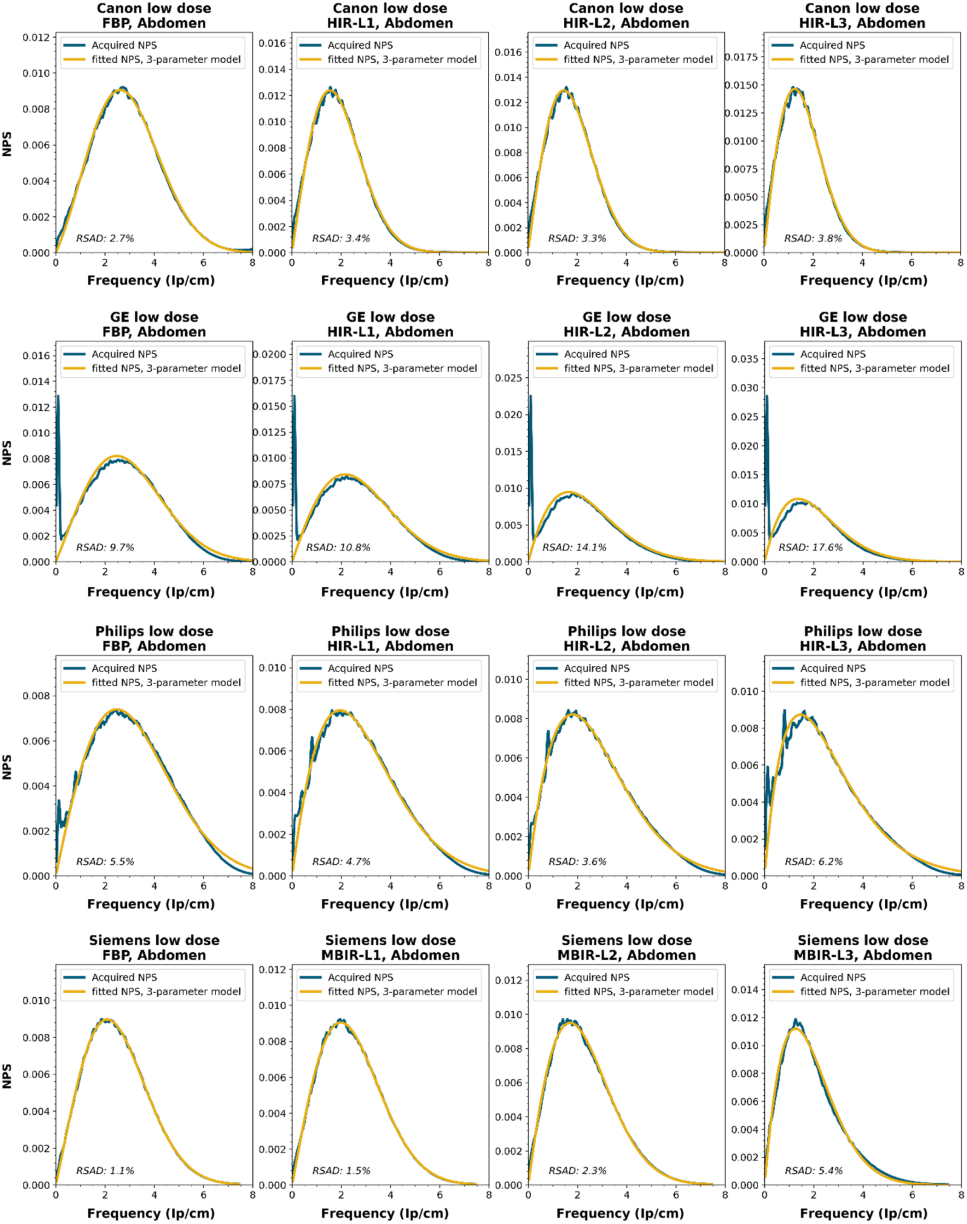


**Figure:**
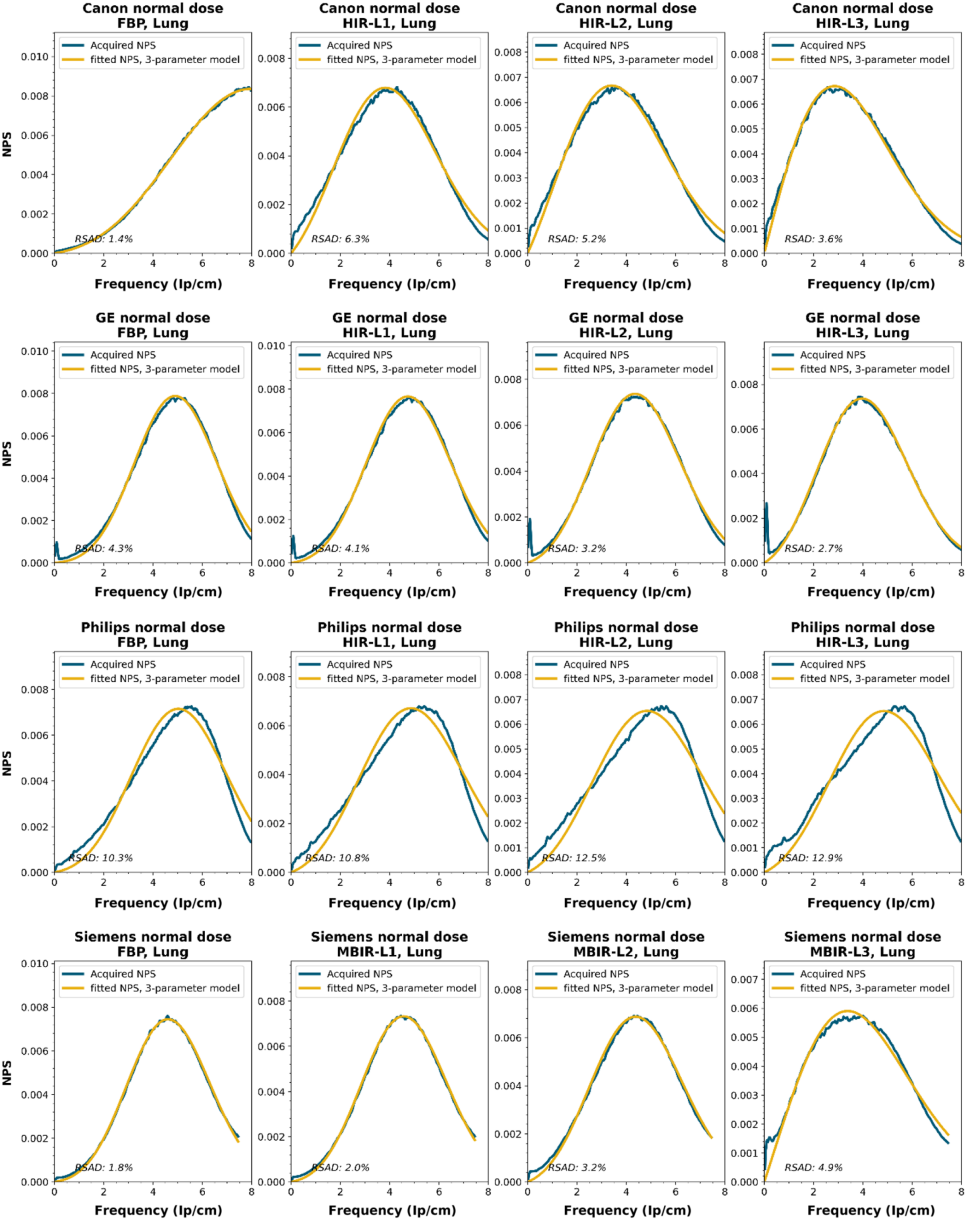


**Figure:**
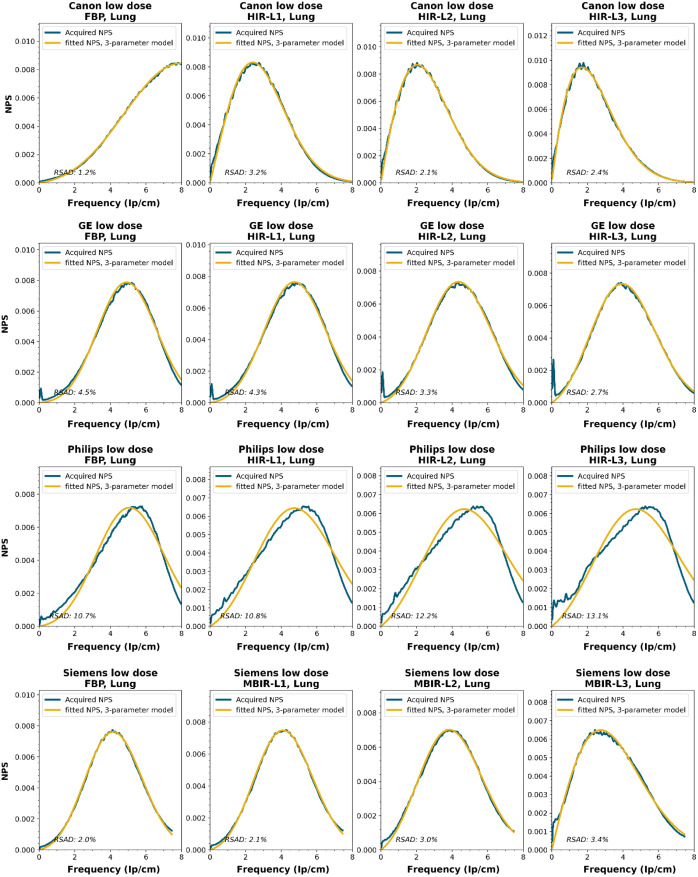


**Figure:**
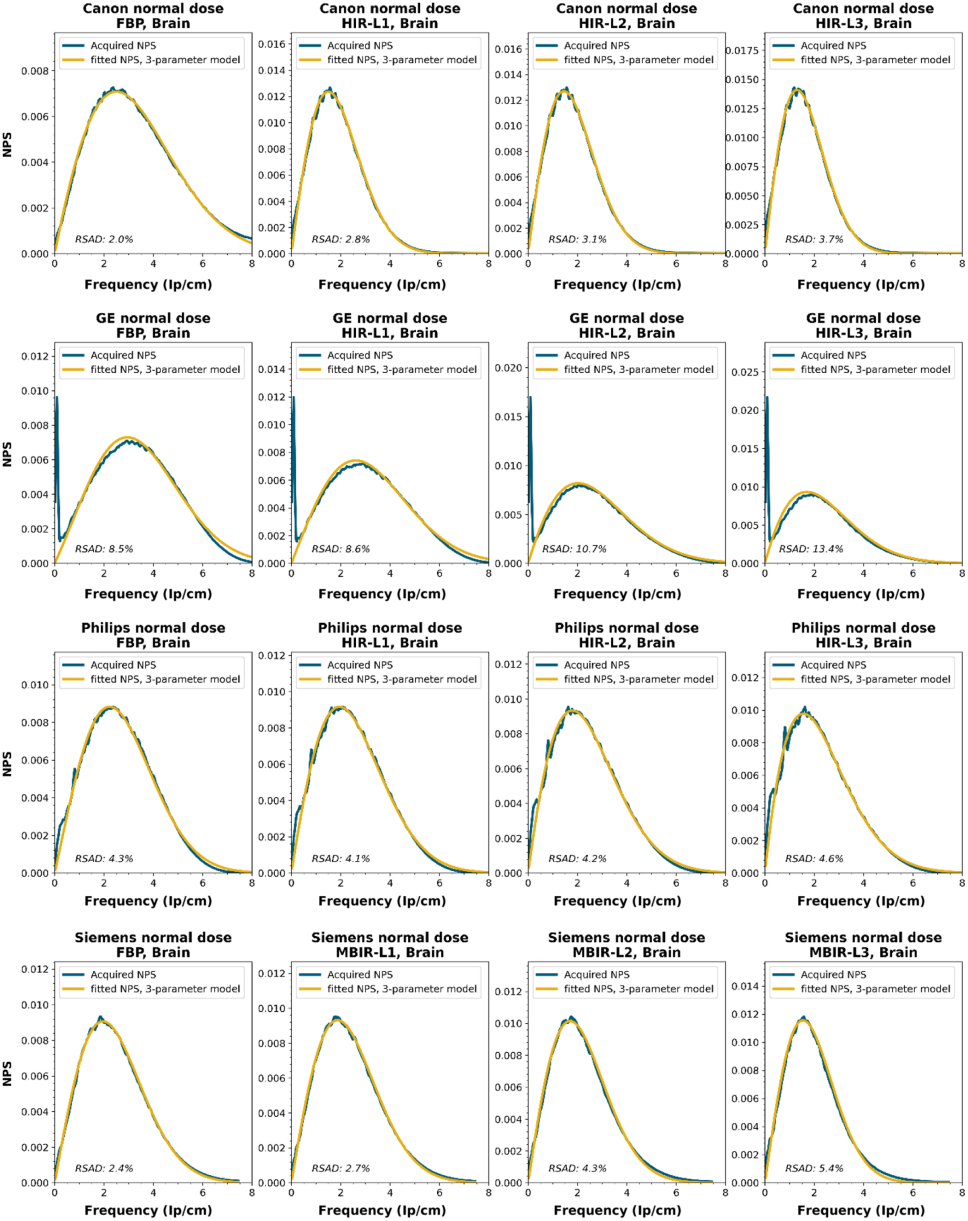


**Figure:**
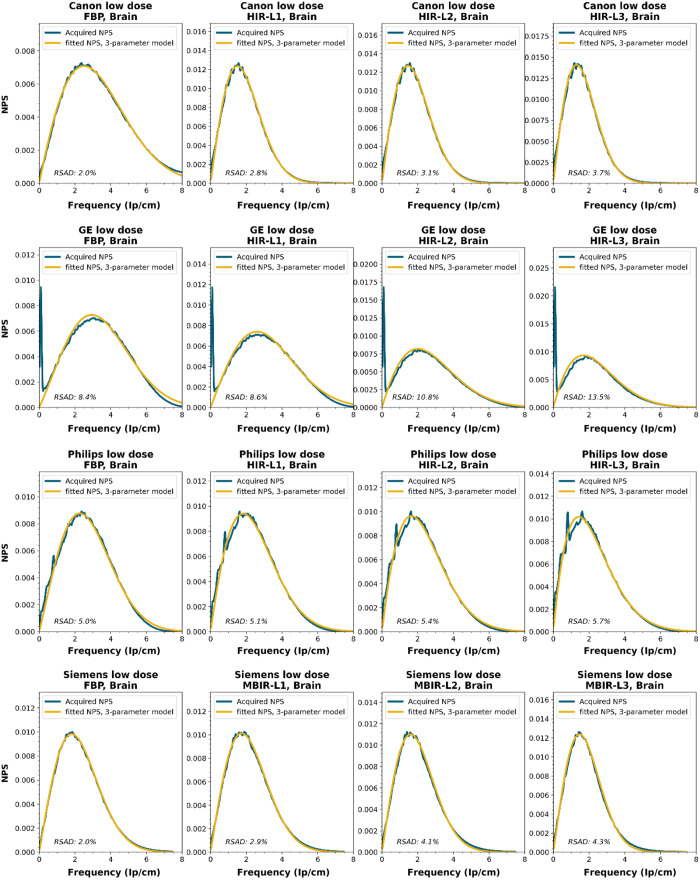


**Figure:**
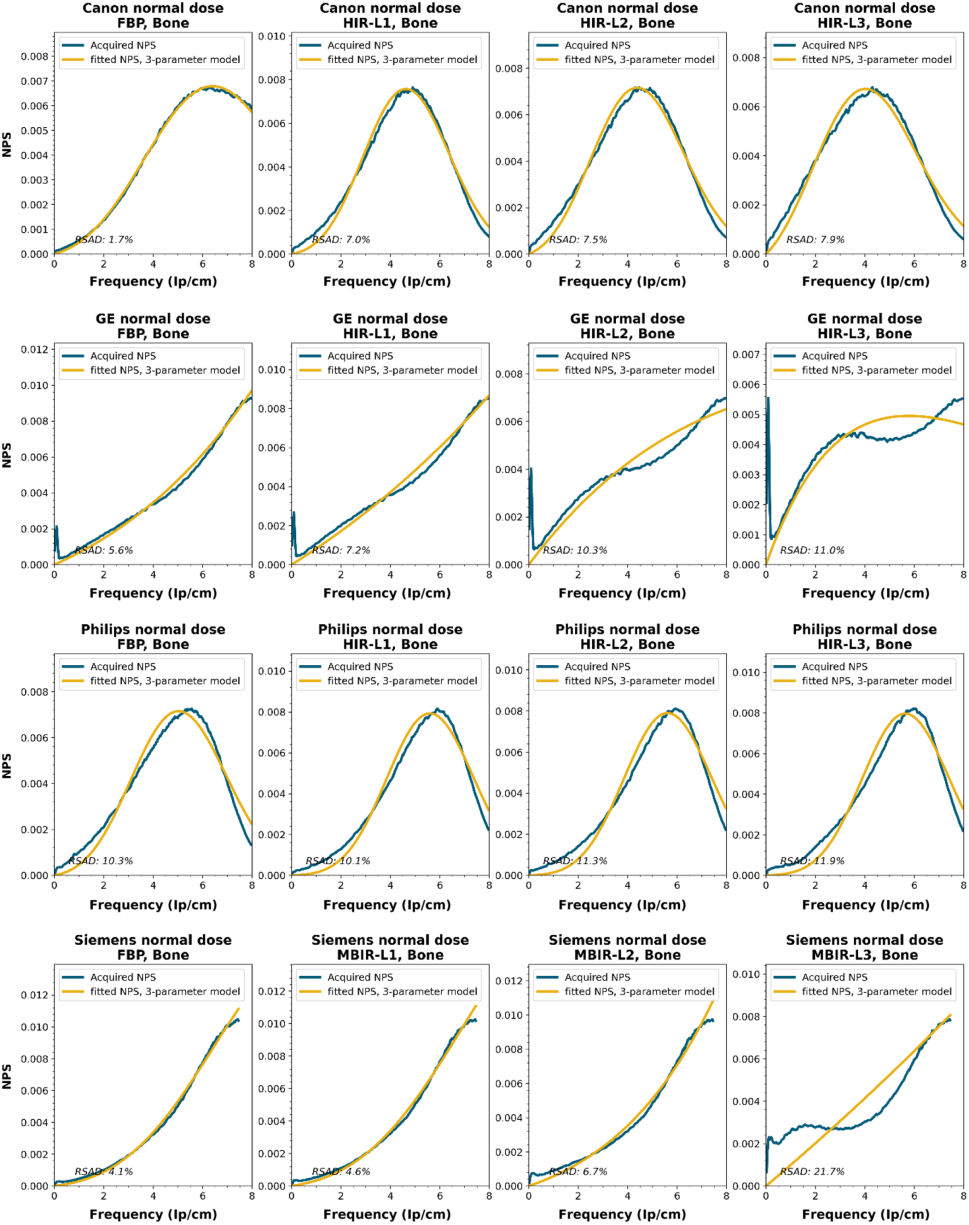


**Figure:**
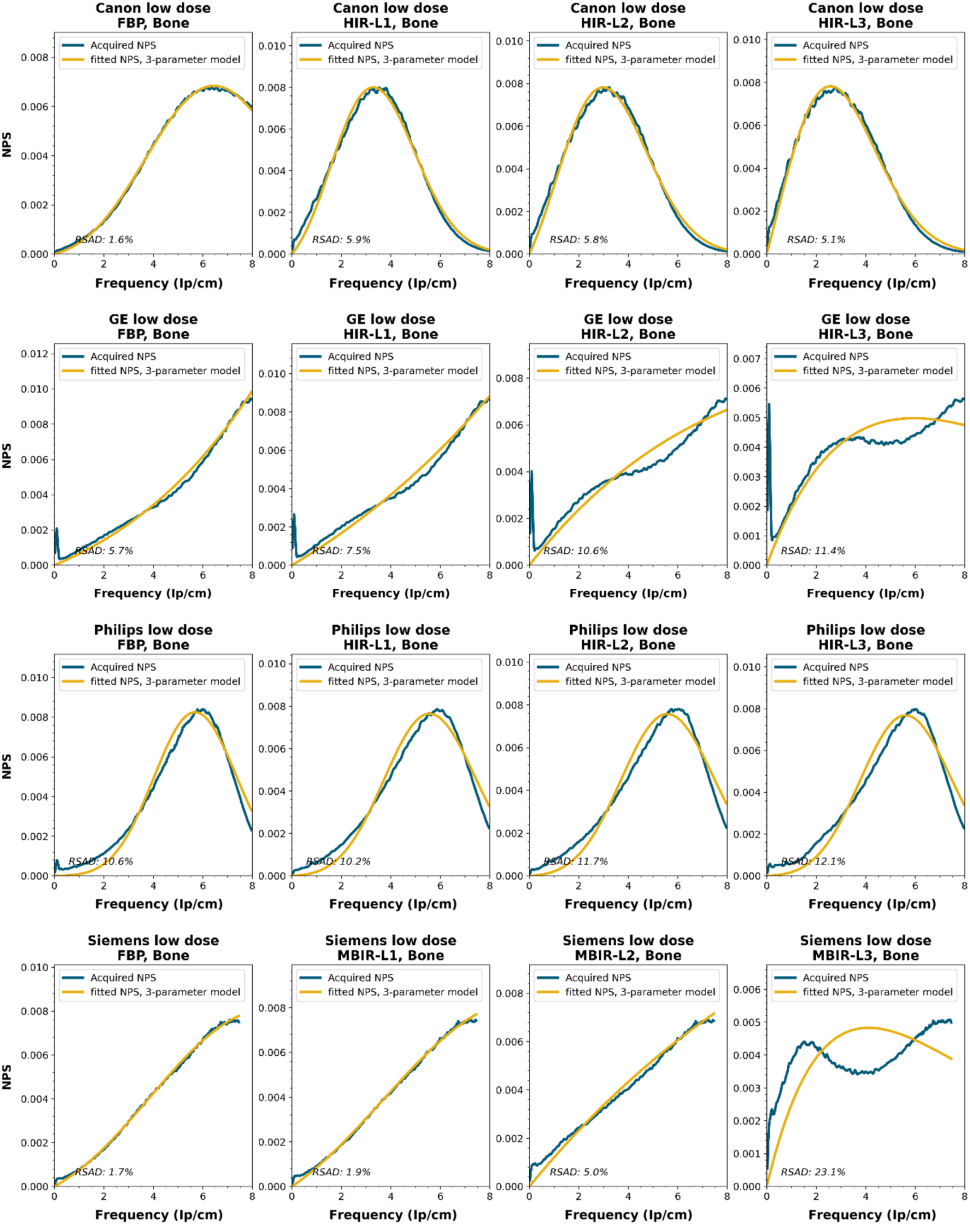


**Figure:**
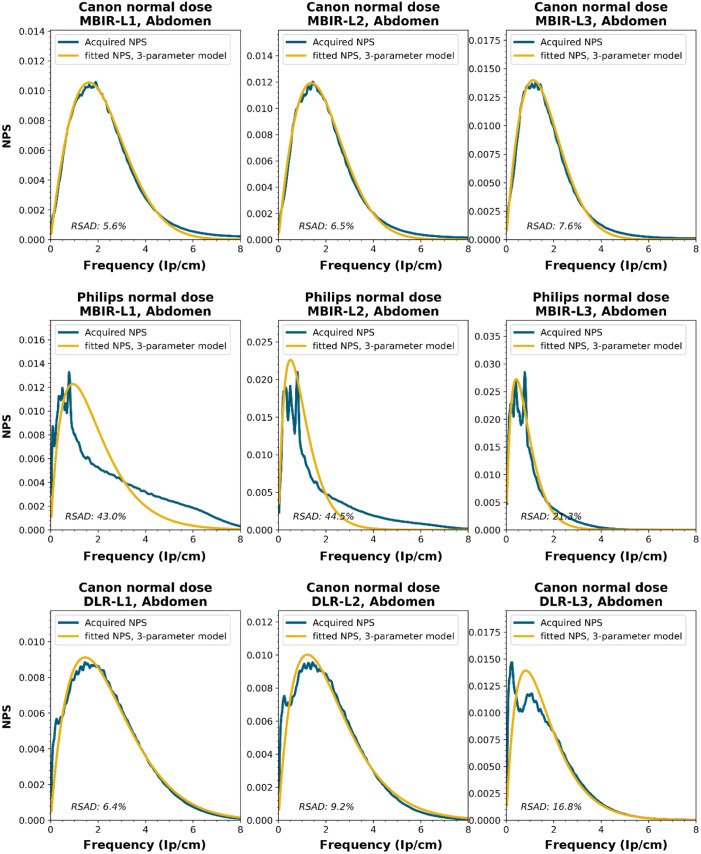


**Figure:**
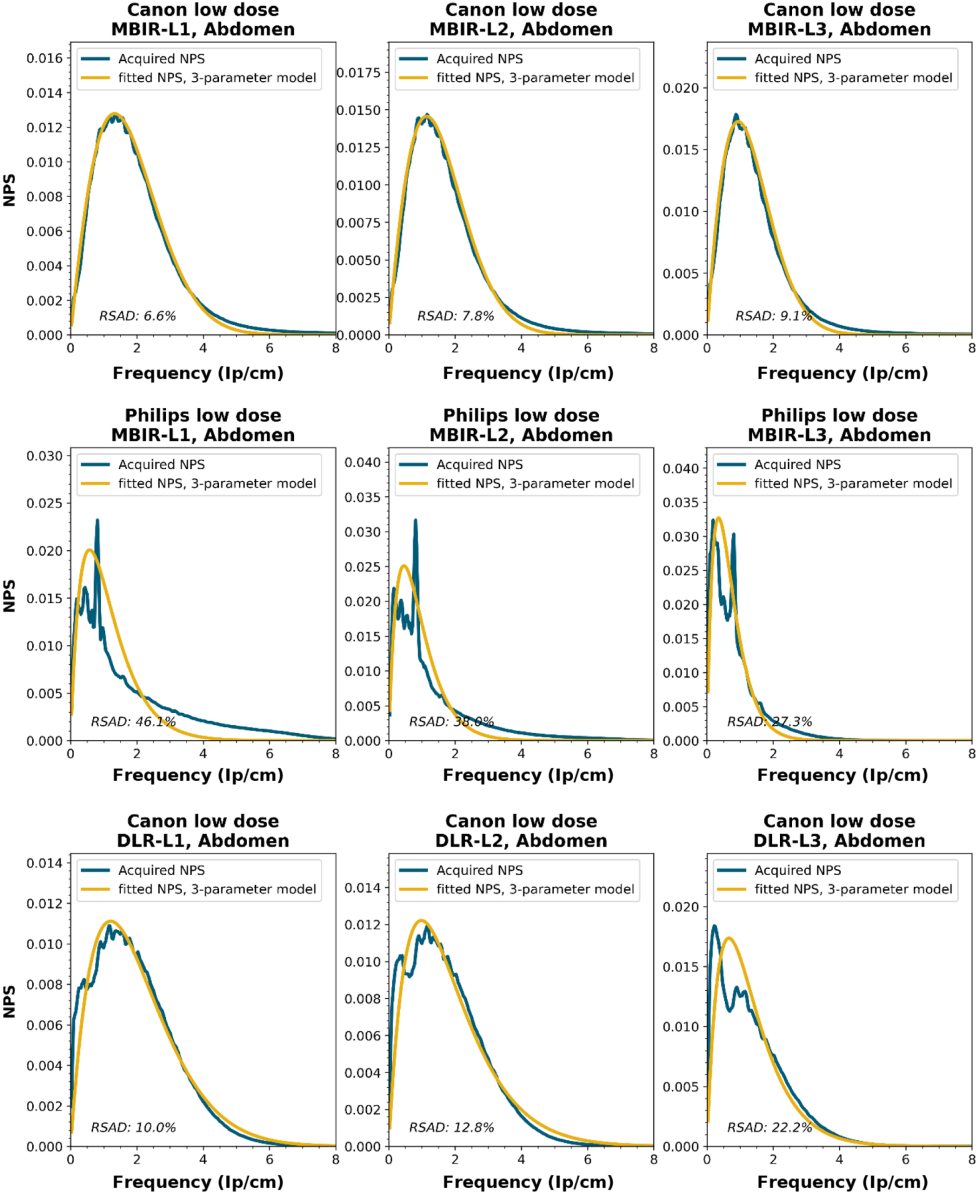


**Figure:**
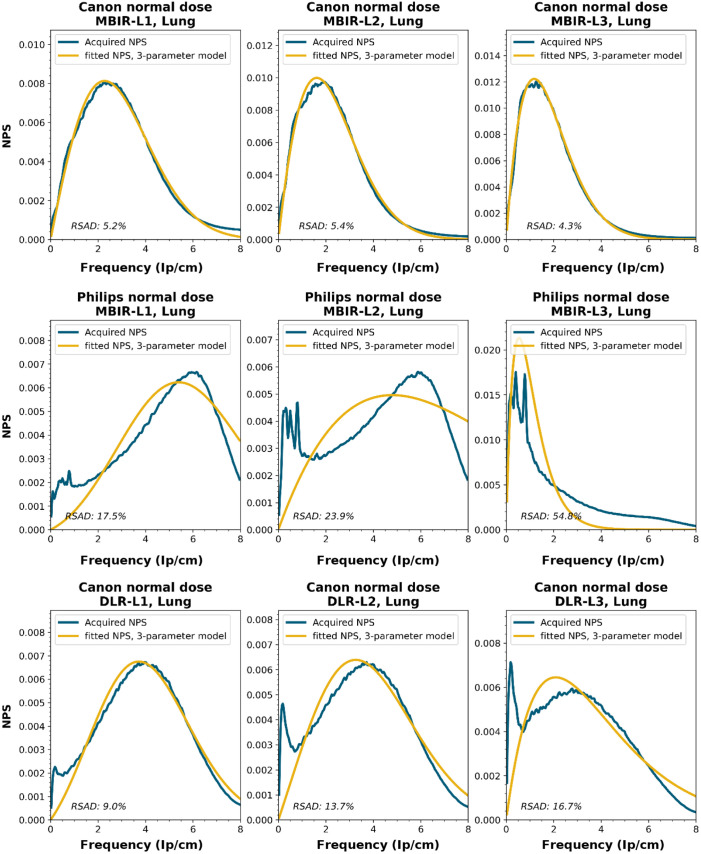


**Figure:**
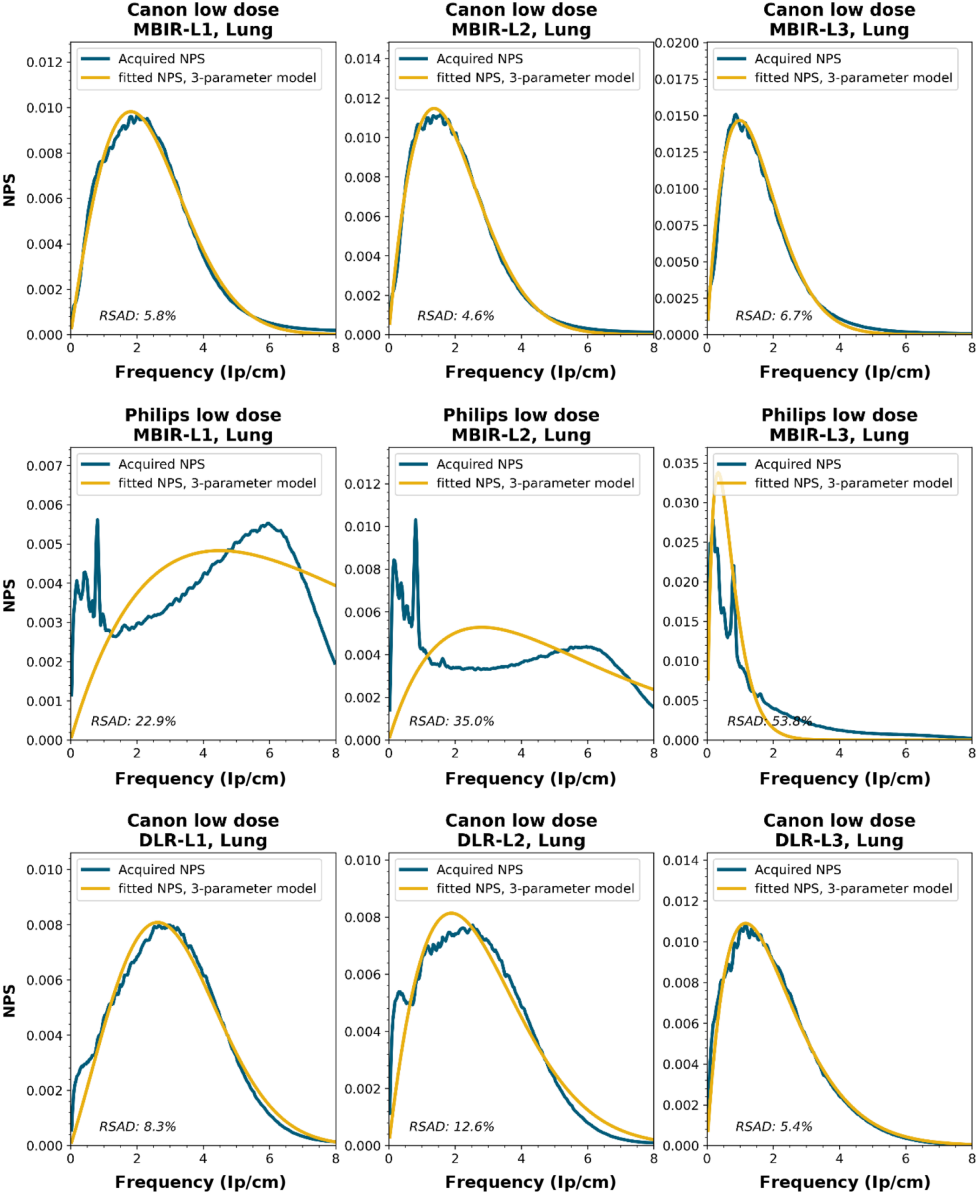


**Figure:**
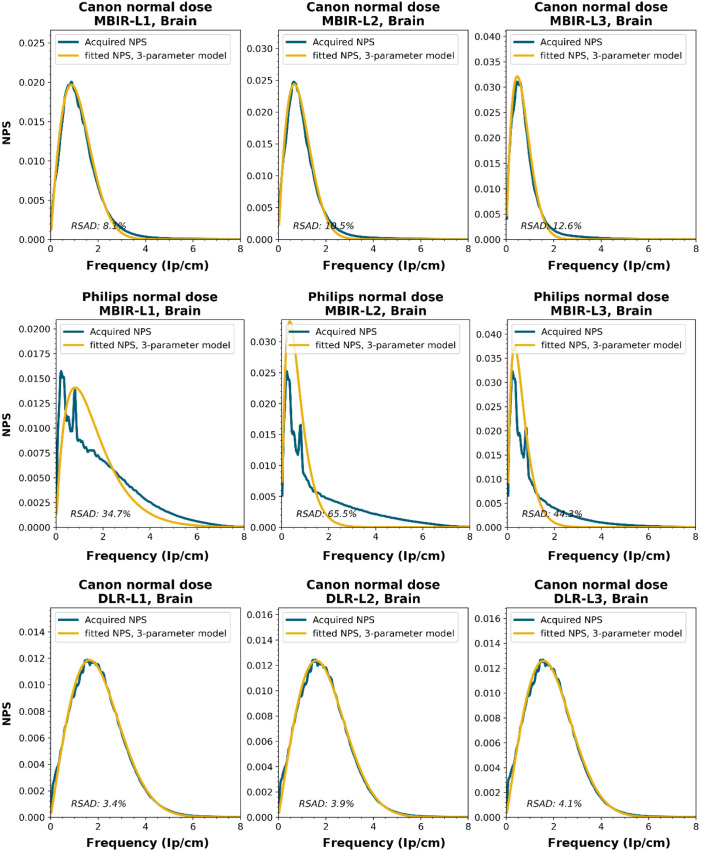


**Figure:**
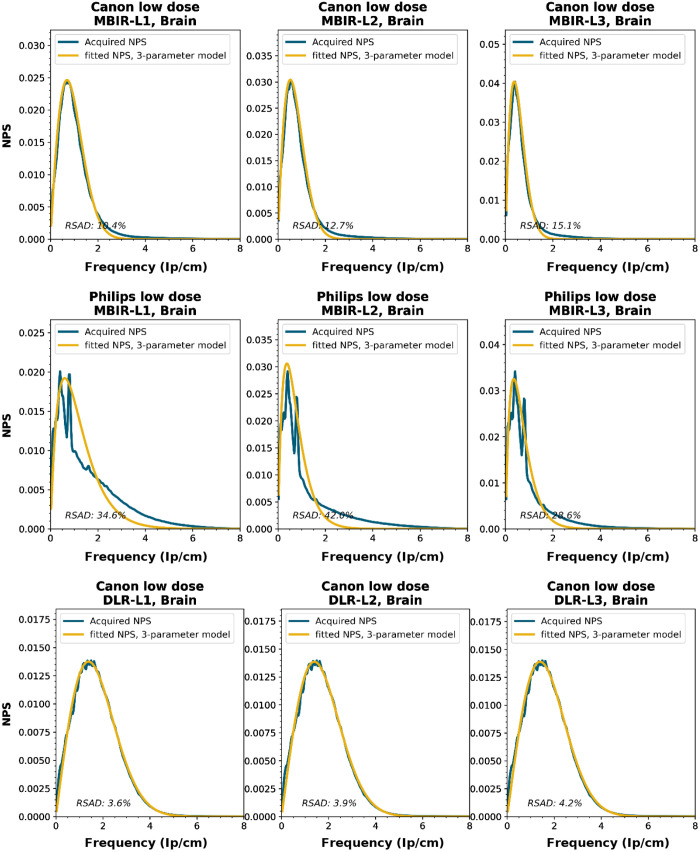


**Figure:**
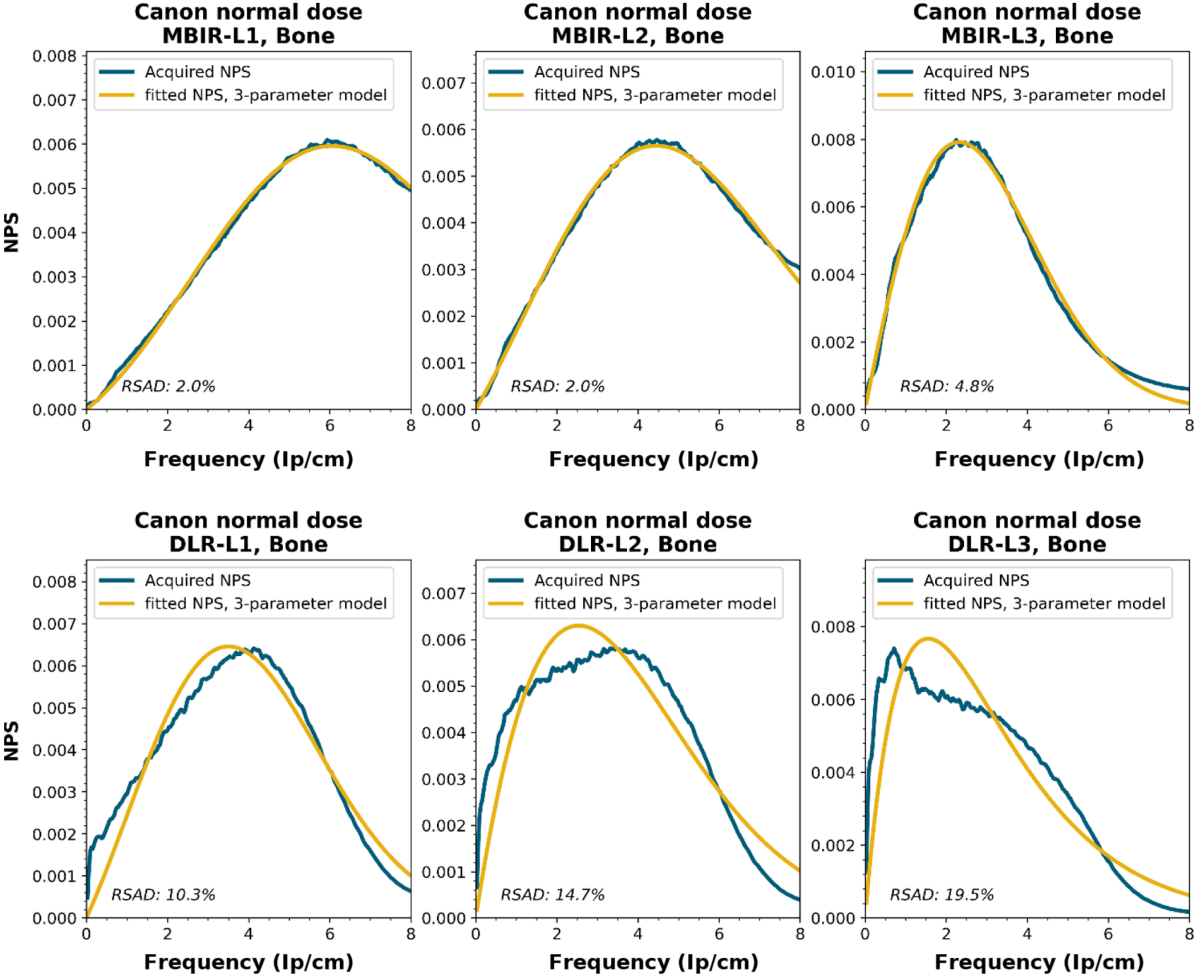


**Figure:**
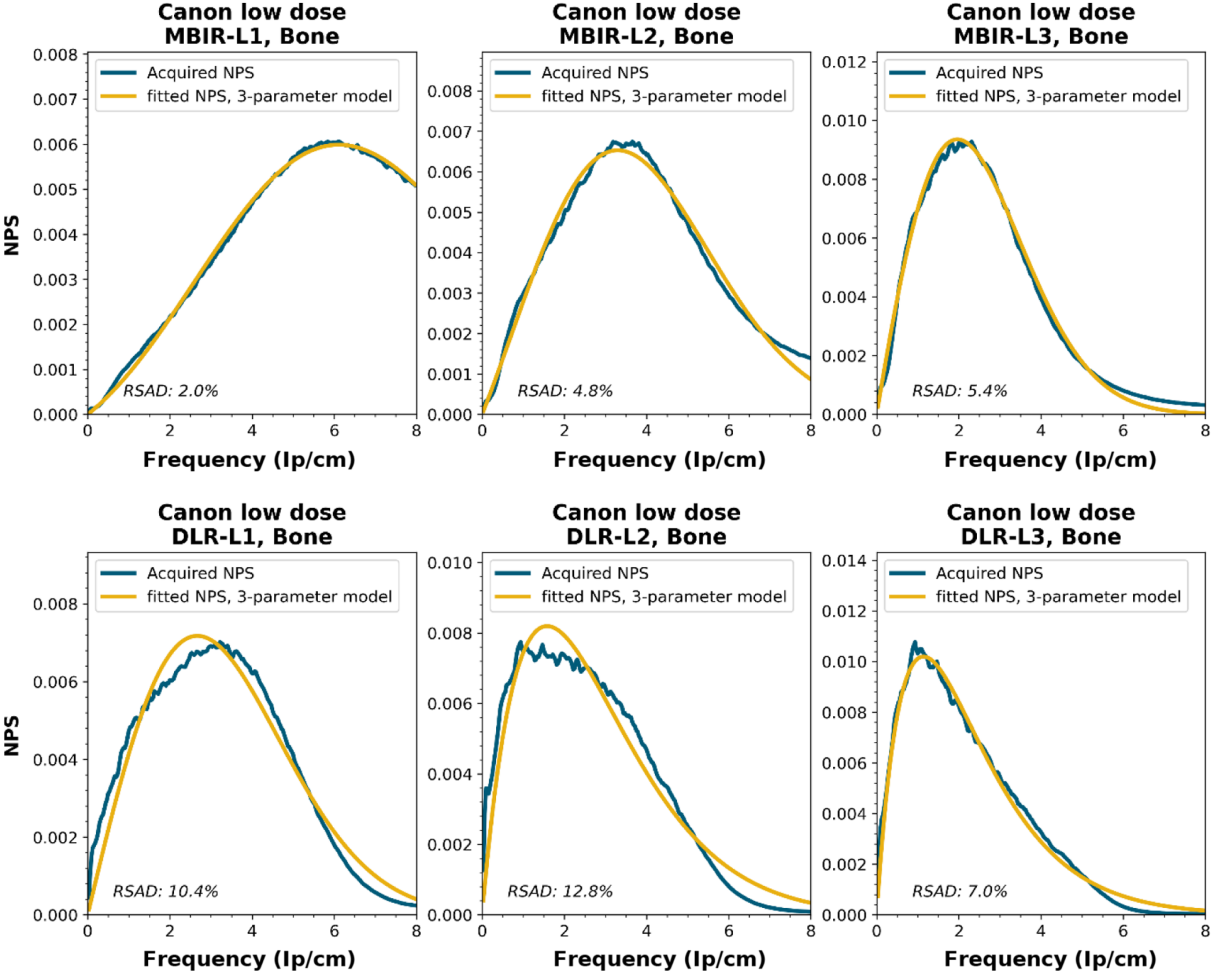


Notes

RSAD: Relative sum of absolute differences

The following reconstruction techniques were not available: Siemens HIR and DLR. GE MBIR and DLR. For Philips MBIR Bone, the hospital uses the same reconstruction kernel as for MBIR Lung.

## 7 Appendix B: Ideal Observer

We consider a binary discrimination task to discriminate between two classes of Gaussian-distributed images defined by different power spectra. Let g represent the image pixel values as a column vector. The two hypotheses for the discrimination task are

$$H_{1}:\;g∼\mathrm{MVN}(\mu ,\Sigma_{1}),\;H_{2}:\;g∼\mathrm{MVN}(\mu ,\Sigma_{2}),$$

where the textural differences are entirely represented in the image covariance matrix ($\Sigma_{1}$ versus $\Sigma_{2}$) and there is no difference in the image mean (μ).

### 7.1 Ideal Observer

The ideal observer test statistic is based on the log-likelihood ratio. The likelihood of a hypothesis (given an image stimulus) is determined from a multivariate normal distribution:

$$p(g|H_{i})=\frac{1}{(2\pi {)}^{M/2}|\Sigma_{i}{|}^{1/2}}e^{-\frac{1}{2}((g-\mu {)}^{T}\Sigma_{i}^{-1}(g-\mu ))},$$

where M is the number of pixels in the image and | | represents the determinant of the matrix argument. Calling this a likelihood (instead of a probability density function) means that we consider g to be given (i.e., the independent variable) and $H_{i}$ to be unknown (the dependent variable). The log-likelihood ratio is then given as

$$\lambda (g)=\log(\frac{p(g|H_{2})}{p(g|H_{1})})\;=-\frac{1}{2}((g-\mu {)}^{T}(\Sigma_{2}^{-1}-\Sigma_{1}^{-1})(g-\mu )+\log(|\Sigma_{2}|)-\log(|\Sigma_{1}|))\;≃(g-\mu {)}^{T}(\Sigma_{1}^{-1}-\Sigma_{2}^{-1})(g-\mu ).$$

The last line is equivalent to the log-likelihood ratio, with removing the terms that do not affect the performance.

To evaluate the ideal observer in this case, we need to be able to compute the inverse of the class covariance matrices. This is where textures defined by an NPS can make the computations much easier.

### 7.2 Frequency Domain Computation

If the different image textures may be considered to be realizations of a stationary random process, then their covariance matrices are diagonalized by the Fourier basis:

$$\Sigma_{i}=F^{-1}S_{i}F↔S_{i}={F\Sigma }_{i}F^{-1},$$

where $S_{i}$ is a diagonal matrix representing the noise power spectrum and F is the finite (usually 2D) Fourier transform matrix. So the product Fg would be the FFT of image g. Because of the properties of the FFT, we have $F^{-1}=\frac{1}{M}F^{*}$, where the superscript * means the transpose conjugate (sometimes called the Hermitian or adjoint operator). So we have

$$S_{i}^{-1}={F\Sigma }_{i}^{-1}F^{-1}\;=\frac{1}{M}{F\Sigma }_{i}^{-1}F^{*}.$$

We can use the spectral decomposition of the covariance matrices in the likelihood ratio to recast the ideal observer formula in the Fourier transform domain. Let the caret represent a Fourier transform (i.e., g^=Fg). We write g−μ as F−1(g^−μ^) to get

λ(g)=(g−μ)*(Σ1−1−Σ2−1)(g−μ)=(F−1(g^−μ^))*(Σ1−1−Σ2−1)F−1(g^−μ^))=1M2(g−μ)*(FΣ1−1F*−FΣ2−1F*)(g^−μ^).

Note that we rewrote $(g-\mu {)}^{T}$ as $(g-\mu {)}^{*}$. This is appropriate because the quantity is real, and therefore the Hermitian is equivalent to the transpose. Based on the formula for the inverse spectrum above, we get

λ(g)=1M(g^−u^)*(S1−1−S2−1)(g^−u^).

Because the power-spectrum matrices are diagonal, their inverses are as well, so this quadratic form can be written as a sum

λ(g)=1M∑k=1M(1S1[k,k]−1S2[k,k])|g^[k]−μ^[k]|2.

This sum can be problematic if any of the spectral elements are 0, which usually happen from power spectra that are estimated from samples. It may be advisable to regularize the power-spectrum inversion to get

λReg(g)=1M∑k=1M(1S1[k,k]+ε2−1S2[k,k]+ε2)|g^[k]−μ^[k]|2,

where ε represents the variance of discretization “noise.”

## Data Availability

All NPS shapes and fits are available in Appendix A of this article as well as all $f_{\text{peak}}$ and σ values. The numeric values of the NPS shapes are available on request and with permission from the hospitals from which these data are retrieved. The code to calculate the fits and the $f_{\text{peak}}$ and σ from an NPS is available at GitHub (https://github.com/radboud-axti/NPS_peak_sigma).
